# Chemistry of 2-(2′-Aminophenyl)benzothiazole Derivatives: Syntheses, Photophysical Properties and Applications

**DOI:** 10.3390/molecules30081659

**Published:** 2025-04-08

**Authors:** Ekaterina K. Pylova, Taisiya S. Sukhikh, Alexis Prieto, Florian Jaroschik, Sergey N. Konchenko

**Affiliations:** 1 Nikolaev Institute of Inorganic Chemistry, Siberian Branch of the Russian Academy of Sciences, 3 Lavrentiev Ave., 630090 Novosibirsk, Russia; e.pylova@g.nsu.ru (E.K.P.); sukhikh@niic.nsc.ru (T.S.S.); 2 Department of Natural Sciences, Novosibirsk State University, 630090 Novosibirsk, Russia; 3 ICGM, Univ Montpellier, CNRS, ENSCM, 34090 Montpellier, France

**Keywords:** heterocycle, benzothiazole, metal–organic compounds, luminescence, ESIPT, sensors, bioimaging, photocatalysis

## Abstract

2-(2′-aminophenyl)benzothiazole is a readily tunable fluorescent core with widespread applications in coordination chemistry, sensing, light-emitting processes, medicinal chemistry, and catalysis. This review provides an overview of the synthetic methodologies to access 2-(2′-aminophenyl)benzothiazole and its organic derivatives, including various phosphorous and silane pincer ligands. The luminescent properties will be discussed, with a special focus on ESIPT and AIE processes. The coordination of transition metals and lanthanides is presented, as well as their influence on biological and light-emitting properties. 2-(2′-aminophenyl)benzothiazole derivatives have also been employed as sensors for a range of cations and anions due to their various binding modes, as well as for bioimaging purposes. Recently, the first application in photocatalysis has emerged, showing one of the many openings for these organic building blocks in the future.

## 1. Introduction

**Figure 1:**
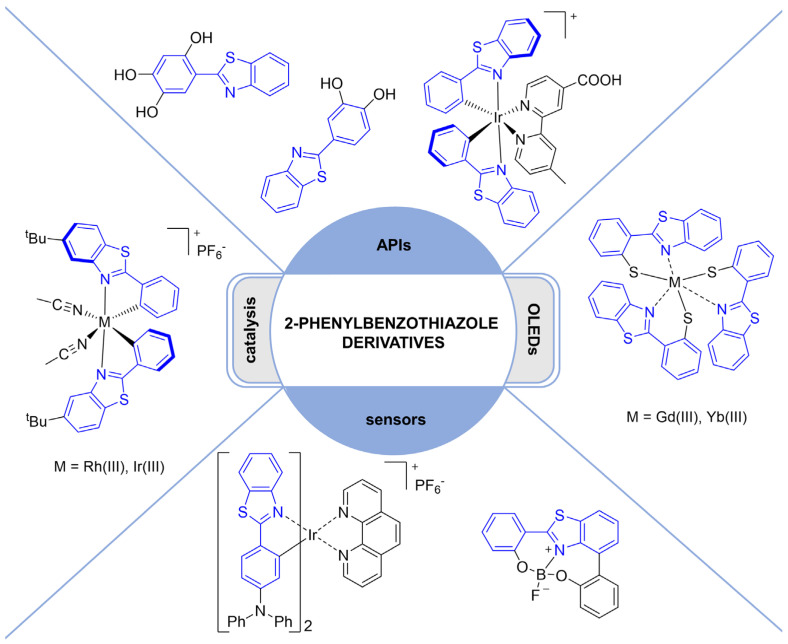
Main applications of ***pbt*** derivatives [45,52,54,56,59,60].

The chemistry of sulfur–nitrogen heterocyclic compounds is one of the main areas of the chemical industry and is involved in nearly all fields of modern life. The developed compounds have significant contributions in molecular and cellular biology [1,2,3,4], medicine [5,6,7,8,9], optoelectronic technologies [10,11,12,13,14,15,16,17,18,19,20,21,22,23], organic field-effect transistors (OFET) [24,25,26], sensors [27,28,29,30], solar cells [31,32,33,34,35], dyes [36,37], and the chemistry of other functional materials [38,39]. Moreover, such compounds have also found applications as fungicides [40,41] or catalysts [42,43,44]. A particularly notable example is the 2-phenylbenzothiazole (***pbt***) fragment, which is an important chromophore group, contributing to the utilization of its derivatives in many practical applications. Derivatives of ***pbt*** are widely used as building blocks in organic and organometallic chemistry due to their interesting photophysical properties, the ease of their synthesis, and the low cost of the starting reagents. The chemistry of ***pbt*** derivatives is a well-studied area that continues to be actively researched: the known organic derivatives include over 120 k compounds, combining amines, amides, alcohols, thiols, phosphines, silicium-, boron-, selenium-containing compounds. The photophysical and electronic properties of these compounds enable their use as ligands in transition metal complexes [45,46,47,48,49,50,51,52], in the biochemical field (bio-probes, antioxidants, etc.) [53,54,55,56,57,58], as light-emitting elements in chemical sensors [59,60], or in luminescent materials (organic light-emitting devices, OLEDs) [61,62,63,64,65] (Figure 1).

The ***pbt*** derivatives bearing different functional groups such as -OH, -SH, or -NHR in the 2′-position of the phenyl group have attracted scientific interest as they often undergo intramolecular proton transfer in the excited state (ESIPT) or display Aggregation-Induced Emission (AIE) phenomenon [66,67,68,69]. These processes are related to hydrogen bonding between the acidic hydrogen of these functional groups and the N atom of the heterocycle. In particular, 2-(2′-aminophenyl)benzothiazole (**2-NH_2_-pbt**, **1**) is a promising core due to the possibility of introducing donor/electron-withdrawing substituents at the amino group. This feature gives ready access to tunable photophysical properties related to ESIPT and/or AIE and also provides additional metal coordination centers [70,71,72,73]. This review will focus on the various synthetic pathways of **1** and its derivatives, as well as on the numerous applications of these emerging organic building blocks in coordination chemistry, sensing, medicinal chemistry, and catalysis.

## 2. Overview of the Properties and Syntheses of 2-(2′-Aminophenyl)benzothiazole Derivatives

### 2.1. Structural Characterizations of 2-(2′-Aminophenyl)benzothiazole ***1*** and Its Derivatives

**Figure 2:**
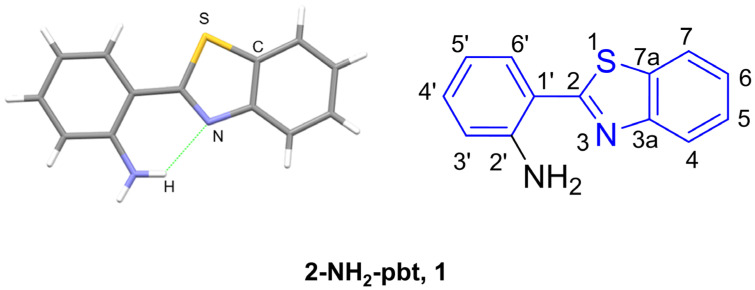
Molecular structure of **2-NH_2_-pbt**, **1** obtained by SCXRD (DMSO solvent not shown) [74].

There are two primary ways of numbering atoms in **2-NH_2_-pbt 1** found in the literature, namely the IUPAC nomenclature and a commonly used alternative, which will be used in this work, as shown in Figure 2.

#### 2.1.1. XRD Analysis

The only reported crystal structure of compound **1** was published as a 1:1 solvate with DMSO (Figure 2), where each solvent molecule is disordered in two positions (not shown in the figure for simplicity) [74]. The two amine molecules in the unit cell exhibit an almost planar structure, with the angle between the benzothiazole plane and phenyl ring in the range of 4.1–5.4° and an intramolecular hydrogen bond between N^2′^-N^3^ (2.68–2.75 Å). Similar planar structures were obtained for closely related 2-(2′-(N-methyl)aminophenyl)benzothiazole (**2**, 3.1–3.5°) [75] and 2-(2′-amino-5′-methylphenyl)benzothiazole (**3**, 3.0°) [76], whereas for other derivatives, including amides, imines, aminophosphines, etc., the angle could reach up to 20° [27,48,50,62,72,77,78,79,80,81,82,83,84,85]. This implies some influence of the substituent at the N atom on the geometry of the ***pbt*** moiety and, as a consequence, on the strength of the hydrogen bond.

#### 2.1.2. NMR Spectroscopy

Compound **1** is an aromatic system in which all proton signals appear in the range of 6.10–8.10 ppm in the ^1^H NMR spectrum. In the ¹³C NMR spectrum, carbons C^2′^, C^2^, and C^3a^, which are bonded to nitrogen atoms, have chemical shifts in the low-field region, while other carbons show signals in the aromatic range. An extensive NMR investigation of 43 2-aryl benzothiazole derivatives was performed in 2006, where one- and two-dimensional NMR experiments were recorded in DMSO-d_6_ to characterize ***pbt*** derivatives [86]. Later, Pierens et al. recorded NMR spectra in solvents with different polarity for imidazoles, oxazoles, and thiazoles and tried to predict the NMR spectra via DFT calculations using B3LYP/6-31G(d) [85]. This approach turned out to be reliable for most solvents, except for the prediction of ^1^H spectra in benzene. Table 1 presents the data for **1** from reference [87]. Venkatachalam et al. performed deuterium exchange experiments by adding D_2_O to a solution of alkyl derivatives 2-AlkNH-pbt (Alk = Me, Et, ^n^Pr, ^n^Bu) in CDCl_3_. As a result, the equilibrium between the deuterated and non-deuterated forms of 2-AlkNH-pbt was observed in the NMR spectra. Based on the chemical shifts for atoms directly involved in intramolecular hydrogen bonding or connected to the amino group in ^1^H, ^13^C, ^15^N, and COSY-spectra, it was concluded that the hydrogen bond between the 2-AlkNH group and N^3^ was a strong interaction [75].

### 2.2. Synthesis of 2-(2′-Aminophenyl)benzothiazole and Derivatives

#### 2.2.1. Parent 2-(2′-Aminophenyl)benzothiazole **1**

**Scheme 1:**
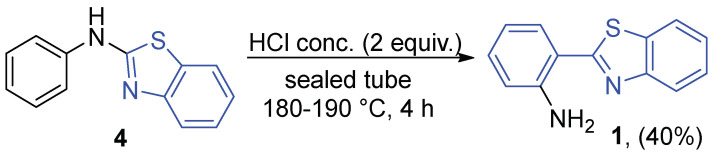
First synthesis of **1** via thermal rearrangement of N-phenyl-2-benzothiazolamine **4** [88].

Meyer et al. first reported the synthesis of **1** in 1934. The rearrangement of N-phenyl-2-benzothiazolamine (**4**) upon heating in a sealed ampoule in the presence of two equivalents of concentrated HCl gave **1** in a 40% yield (Scheme 1) [88].

**Scheme 2:**
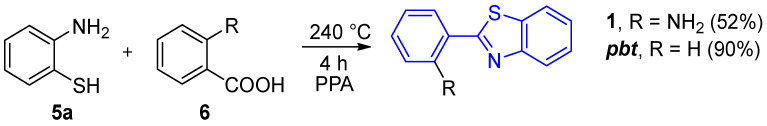
Synthesis of **1** using anthranilic acid **6** as a starting material [89].

For the efficient synthesis of compound **1**, the most commonly used starting compound is 2-aminothiophenol (**5a**), which typically undergoes a condensation reaction with anthranilic acid (**6**). Hein et al. first developed a simple method for synthesizing heterocyclic compounds, including **1** and ***pbt*** [89]. They used polyphosphoric acid (PPA) as both a dehydrating agent and a solvent (Scheme 2), facilitating the intermolecular condensation of various compounds. In the reaction between **5a** and **6**, the yield of **1** was 52%. They carried out the condensation reaction at a high reaction temperature to avoid side reactions. However, it was later shown that decreasing the reaction temperature from 250 °C to 150 °C does not affect the product yield [84,90]. This synthetic route later became one of the most popular and is actively used in contemporary research [77,86,87,91,92,93,94].

Instead of using acid **6** as a starting material, the corresponding aldehyde, anhydride, or even alcohol can be used as well, as described below. Under the reaction conditions, **6** is formed in situ, and further, the condensation reaction takes place, giving the desired amine.

Another widely used approach to synthesize **1** is to use isatoic anhydride (**7**) as a starting material under various reaction conditions. Gajare et al. proposed a method which includes using natural kaolinitic clay as a catalyst. Compound **7** reacted with **5a** in dried chlorobenzene at 120 °C, yielding 55% of **1** (Table 2, entry 1) [95]. Fadda et al. also used **7** in glacial acetic acid with sodium acetate as a catalyst, forming the corresponding anthranilamide (**8**), which then underwent ring closure to yield **1** with an 80% yield (Table 2, entry 2) [96]. Finally, Tseng et al. provided a methodology that produced a 68% yield of **1** using ZnCl_2_ as a catalyst (Table 2, entry 3) [97], although it was later shown that the reaction could proceed without a catalyst with good yields [55,98,99,100,101].

The use of 2-aminobenzaldehyde (**9a**) was applied in several synthetic methods. Ray et al. developed an eco-friendly catalytic way for synthesizing ***pbt*** derivatives in water. The authors modified mesoporous silica material, introducing additional 2-(piperazin-1-yl)pyrimidine functionalized organosilane tails. These tails were responsible for thiolate formation and ring closure steps in the reaction mechanism. The reaction was performed using 2-aminothiophenol disulfide (**10**) and substituted **9a** in a 1 to 2 ratio under O_2_ at 100 °C for 4 h (Table 3, entry (i)). For compound **1**, the reaction yield achieved 89%, while for other ***pbt*** derivatives, it was in the range of 85–98% [102].

In 2016, Basha et al. utilized a microwave-assisted method for the synthesis of **1** (Table 3, entry (ii)). The reaction involved irradiation of **5a**, aldehyde **9a**, and catalytic amounts of glacial acetic acid for 10 min to afford the product in quantitative yield [103].

**Scheme 3:**
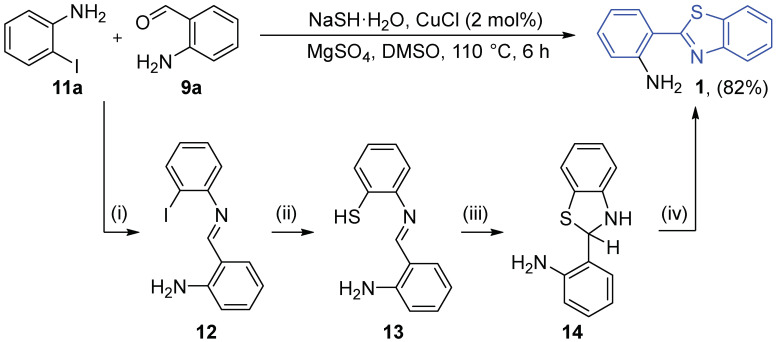
Synthesis of **1** using NaSH as a sulfur source with major steps (i)–(iv) of proposed mechanism [104].

An alternative synthetic method using a different sulfur source was developed by Park et al. They described a one-pot reaction using substituted iodoanilines **11**, aromatic benzaldehydes **9**, and NaSH·nH_2_O as a sulfur source, leading to the synthesis of ***pbt*** derivatives with good yields (Scheme 3), even with fluoro-, chloro-, and trifluoromethyl substituents in the 2-benzothiazole (***bt***) fragment. In this procedure, which was the first example of using NaSH as a sulfur source for forming C-S bonds, CuCl (2 mol%) was used as a catalyst in DMSO at 110 °C for 6 h, providing **1** in a high 82% yield [104]. From a mechanistic point of view, the reaction would start with the condensation of **11a** and **9a,** affording imine **12** (Scheme 3, step (i)). Then, the copper catalyst would promote the reaction between NaSH·*n*H_2_O with **12**, giving thiophenol **13** (Scheme 3, step (ii)). Subsequently, intramolecular condensation to **14** (Scheme 3, step (iii)) and dehydrogenation would provide the final product **1** (Scheme 3, step (iv)).

**Scheme 4:**
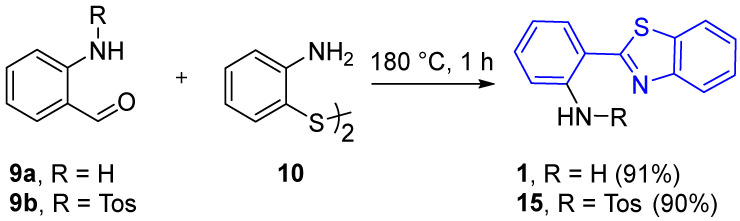
Synthesis of **1** and **15** under molten state reaction conditions [105].

Bakthadoss et al. successfully synthesized about 45 heterocyclic compounds using a molten state reaction, which did not require the use of a catalyst or solvent. Compound **1** and 2-(2′-(N-tosyl)aminophenyl)benzothiazole (**15**) were obtained in excellent yield from the reaction of **10** with the corresponding aldehyde (**9a** or **9b**) at 180 °C (Scheme 4) [105].

A straightforward method of synthesizing amino derivatives in organic chemistry is via a sequential strategy (Table 4) involving the chemical reduction of the corresponding nitro compound. Several groups have used this approach [28,106,107,108], introducing 2-nitrobenzaldehyde **16a** (Table 4, entries 1–3) or nitrobenzoyl chloride **16b** (Table 4, entry 4) into the reaction with **5a**. The isolated 2-(2′-nitrophenyl)benzothiazole (**17**) was then reduced using common reducing agents such as Sn(II), Fe(0) in an acidic medium, or hydrazine. Compound **1** was formed with good yields ranging from 70 to 85%.

Another catalytic approach for the synthesis of heterocyclic compounds involved the use of 2-aminobenzyl alcohol (**18**). Shi et al. utilized substituted primary alcohols **18**, **5a,** and a catalytic quantity of NaO^t^Bu (20 mol%) for synthesizing a series of ***bt*** derivatives. The reaction was carried out in toluene at 100 °C for 24 h using an air balloon (Table 5, entry 1). The authors demonstrated the versatility of the methodology on more than 50 benzazoles in mostly good-to-excellent yields. However, this method gave only 43% of the reaction yield in the case of **1** [109].

**Scheme 5:**
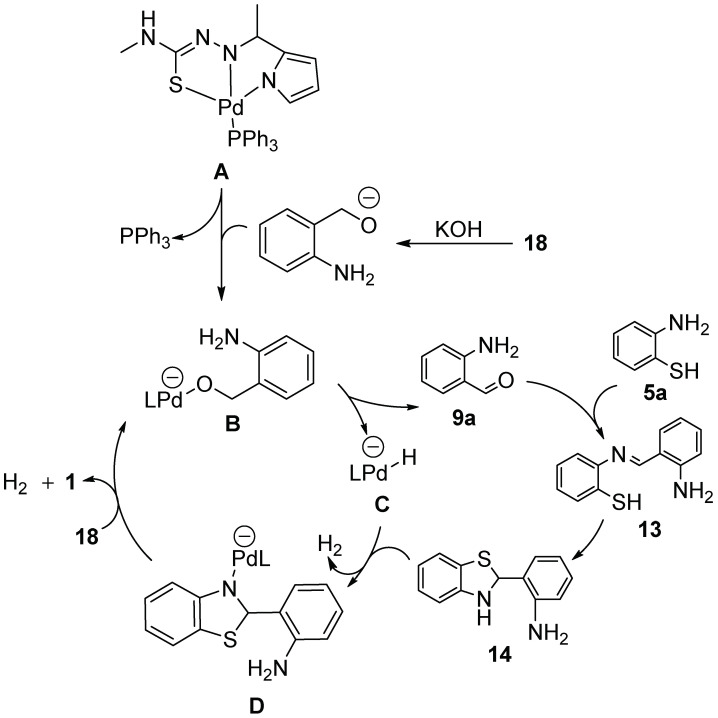
Plausible mechanism for the synthesis of **1** from 2-aminobenzyl alcohol **18** involving a Pd(II) catalytic system [110].

Anandaraj et al. introduced a palladium-catalyzed approach for the synthesis of 25 ***pbt*** derivatives [110]. The pincer-ligand bearing Pd(II) complex **A** was employed in the acceptorless dehydrogenative coupling strategy (Table 5, entry 2). In the plausible reaction mechanism (Scheme 5), base treatment of benzyl alcohol **18** would result in the formation of an alkoxide, which would displace triphenylphosphine from palladium complex **A,** affording new complex **B.** Dehydrogenation in **B**, would lead to the hydride complex **C** and aldehyde **9a**. The latter would react with **5a,** yielding imine **13**, followed by rearrangement into **14**. Subsequent reaction of heterocyclic compound **14** with hydride complex **C** would provide Pd-complex **D**, which would rapidly dehydrogenate to produce **1** and regenerate complex **B** upon coordination of another alkoxide.

**Scheme 6:**
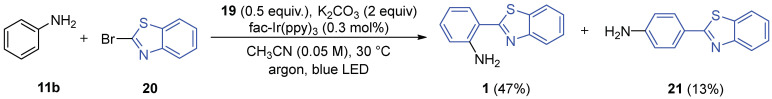
Photocatalytic method for synthesizing a mixture of **1** and **21** using 2-bromoazole **20** [111].

In 2016, a photocatalytic method for obtaining ***bt*** derivatives was described. In this case, a reactive 2-azolyl radical, generated from 2-bromoazole **20** under light irradiation in the presence of an iridium sensitizer and N,N-diisopropyl-N-ethylamine (**19**), reacted with various arenes. Whereas the reaction selectivity was excellent for most compounds, in the case of aniline (**11b**), the authors obtained a mixture of two amines, with the amino group placed in ortho- and para-position to the benzothiazole. The total yield of the two isomers **1** and **21** was 60%, which could be separated using column chromatography (Scheme 6) [111].

**Scheme 7:**
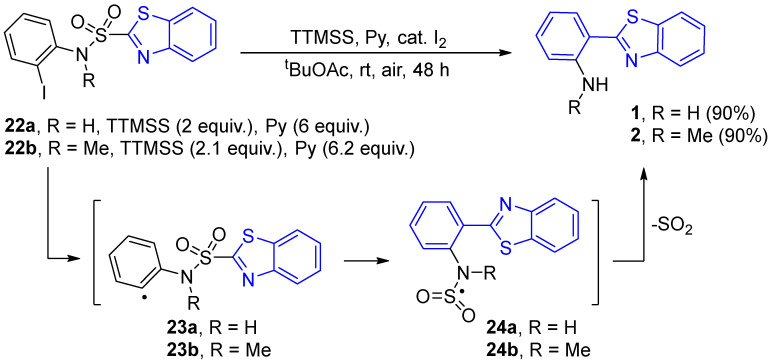
Synthesis of **1** and **2** through an aryl radical transfer mechanism [112].

Nacsa and Lambert described a biaryl rearrangement under mild conditions (Scheme 7), which included the synthesis of ***pbt*** derivatives. Sulfonic acid derivatives were used as starting materials in these cross-coupling reactions in the presence of tris(trimethylsilyl)silane (TTMSS), pyridine, and a catalytic amount of iodine. The proposed reaction mechanism was based on the intramolecular transfer of an aryl radical generated at room temperature via a combination of TTMSS and air. Ten ***pbt*** derivatives were isolated, with yields ranging from moderate to good. In the case of compounds **1** and **2**, the reaction yield reached 90% [112].

**Scheme 8:**
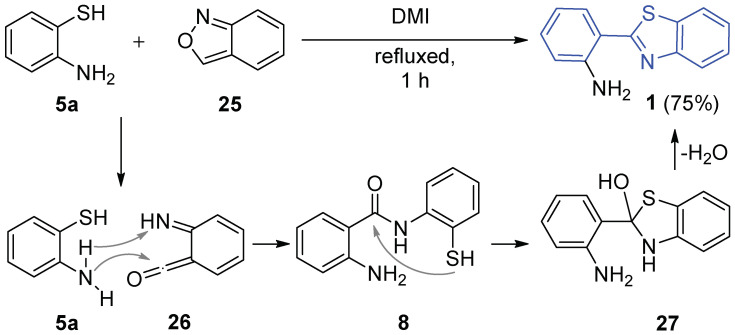
Synthesis of **1** involving 2,1-benzisoxazole **25** [113].

2,1-benzisoxazole (**25**) was used as a starting reagent for the synthesis of **1** (Scheme 8). The authors found that **25** converted into a highly reactive ketene **26** upon refluxing in dimethyl-2-imidazolidinone (DMI), which then reacted with **5a** to anthranilamide **8** and further to **1** with a good yield of 75% [113].

#### 2.2.2. Derivatives of 2-(2′-Aminophenyl)benzothiazole

**Scheme 9:**
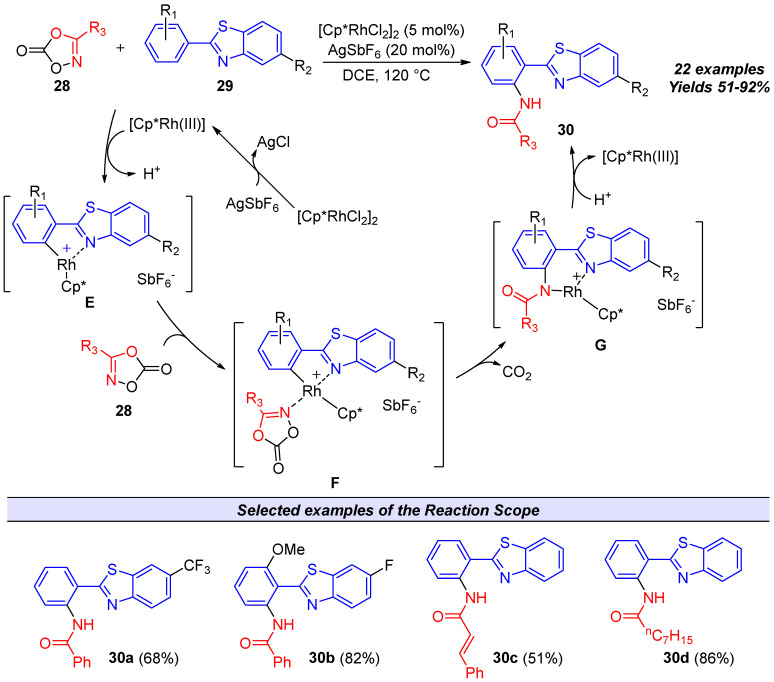
Synthesis of 2-RNH-pbt derivatives **30** from dioxazolone **28** via a Rh-catalyzed approach [62].

In particular cases, the syntheses of amino derivatives of heterocyclic compounds from parent amine can be challenging due to the relatively high stability and low reactivity of the heterocycle, unfavorable chemical reactions, chemical rearrangements, etc. As a consequence, the direct synthetic methods for these derivatives can also be helpful. Among several catalytic works reported in the literature, the Rh-catalyzed C−H amidation approach developed by Liu et al. enabled the synthesis of a series of amides **30** with various substituents either in the benzothiazole ring or in the phenyl group (Scheme 9). From various dioxazolone derivatives (**28**) and substituted 2-phenylbenzothiazoles (**29**) as starting materials, 22 amido derivatives **30** were isolated in moderate-to-high yields. The reaction was proposed to proceed through the in situ generation of a rhodium active species [Cp*Rh(III)], which would react with **29**, giving a cyclometalated rhodium intermediate **E**. Next, dioxazolone derivative **28** would coordinate with the metal center, yielding **F**. The release of carbon dioxide would lead to the rearrangement of the coordination sphere of the rhodium center, facilitating N-C bond formation. Finally, the protonation of intermediate **G** would afford the desired product **30** [62].

**Scheme 10:**
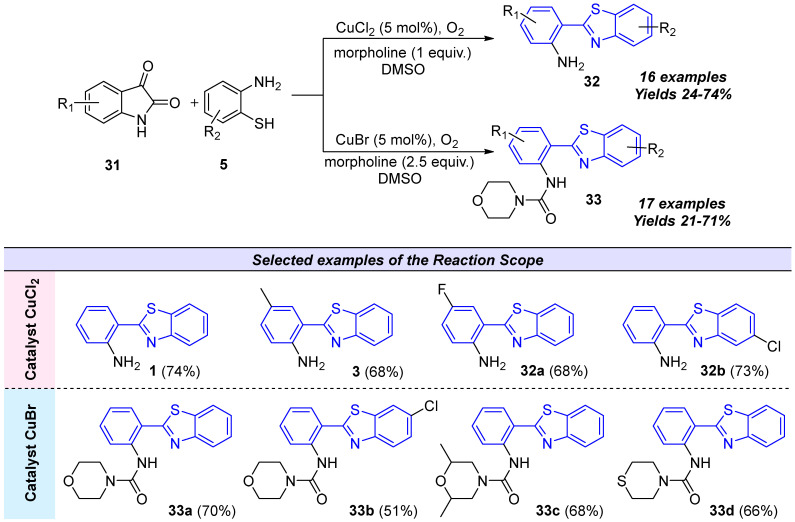
Selective synthesis of 2-aminopbt derivatives **32** and **33** using isatin **31** as a starting material [79].

The selective strategy for the synthesis of a series of ***pbt*** derivatives was proposed by Wang et al. They loaded isatins (**31**) into a sealed tube and heated it at 130 °C with 2-aminothiophenols (**5**) in DMSO in the presence of copper-catalyst and morpholine as a reaction promoter (1 equiv.) or reagent (2.5 equiv.) (Scheme 10). The utilization of CuCl_2_ led to the formation of 2-amino-pbt derivatives **32**, particularly the reaction yield achieved 74% for **1** and 68% for **3**. The CuBr catalyst facilitated further proceeding to a three-component reaction with morpholine, affording urea derivatives **33** in variable yields [79].

**Scheme 11:**
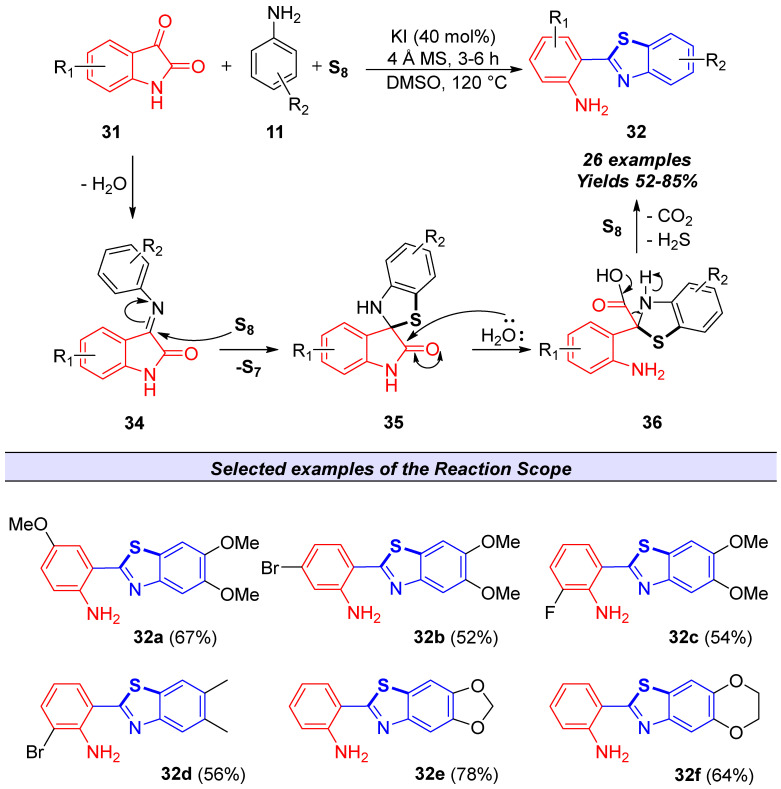
Synthesis of 2-aminopbt derivatives **32** via a metal-free approach from isatin **31** and S_8_ [114].

Isatin (**31**) was also used as a precursor for the synthesis of amino-pbt derivatives **32** in another catalytic approach. Singh et al. introduced this compound into a metal-free reaction with substituted anilines (**11**) and S_8_ as a sulfur source [114]. The reaction was performed in DMSO for 3–6 h in the presence of potassium iodide (40 mol%) as a catalyst, and a set of compounds **36** was isolated in good to high yields (Scheme 11). The only limitation of this method was the para-halide-substituted anilines, which did not react under these conditions. Mechanistically, the reaction of **31** with **11** would lead to the formation of imine **34**, followed by nucleophilic attack by S_8_, resulting in C-S bond formation. Next, an intramolecular electrophilic attack of the aromatic ring on S_8_ would generate the spirocyclic intermediate **35** with loss of S_7_ oligomer. Subsequent nucleophilic addition of H_2_O, followed by C-N bond cleavage of the isatin part, would provide intermediate **36**. Finally, decarboxylation and dehydrogenation would yield products **32**.

**Scheme 12:**
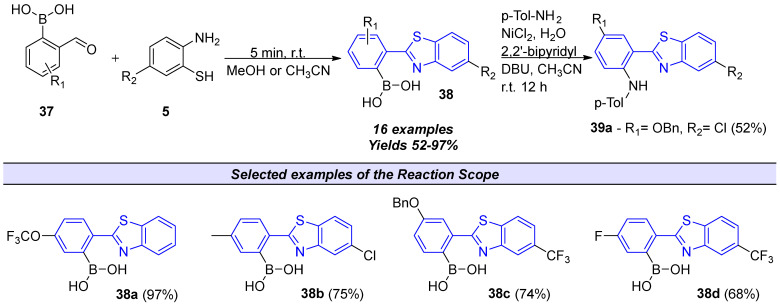
Alternative pathway for synthesis of pbt derivatives **38** and **39** using a click chemistry approach [115].

Click chemistry was shown to be another straightforward method for obtaining 2-amino-pbt derivatives. Draganov et al. applied the boronic acid group both as a catalyst and as a site for further potential functionalization [115]. Aldehydes **37** with a boronic acid group in ortho-position reacted with substituted 2-aminothiophenols **5** (Scheme 12); the full reaction conversion to compound **38** was reached within only 5 min at r.t. The authors showed the versatility of the approach and the possibility to perform the coupling methodologies, such as the formation of C-C or C-N bonds, including the synthesis of the functionalized 2-amino-pbt derivative **39a**.

In conclusion, even though a large range of synthetic methods has been developed, the most convenient and simplest route to **1** is the condensation/cyclization reaction between 2-aminothiophenol **5a** and either a carboxylic acid **6** or a benzaldehyde **9a**, giving the desired product on a gram-scale in moderate to good yields. It is also worth noting that the copper-catalyzed synthetic method (Scheme 10) and the pathways involving isatins as a starting material (Scheme 11) allow for the preparation of derivatives of **1** on a 6 to 60 mmol scale. In some cases, catalytic reactions can be advantageous for synthesizing more complex derivatives. Further functionalization of **1** can be achieved using organic chemistry approaches, as will be described in the following section.

#### 2.2.3. General Pathways Toward Functionalization of 2-(2′-Aminophenyl)benzothiazole

**Figure 3:**
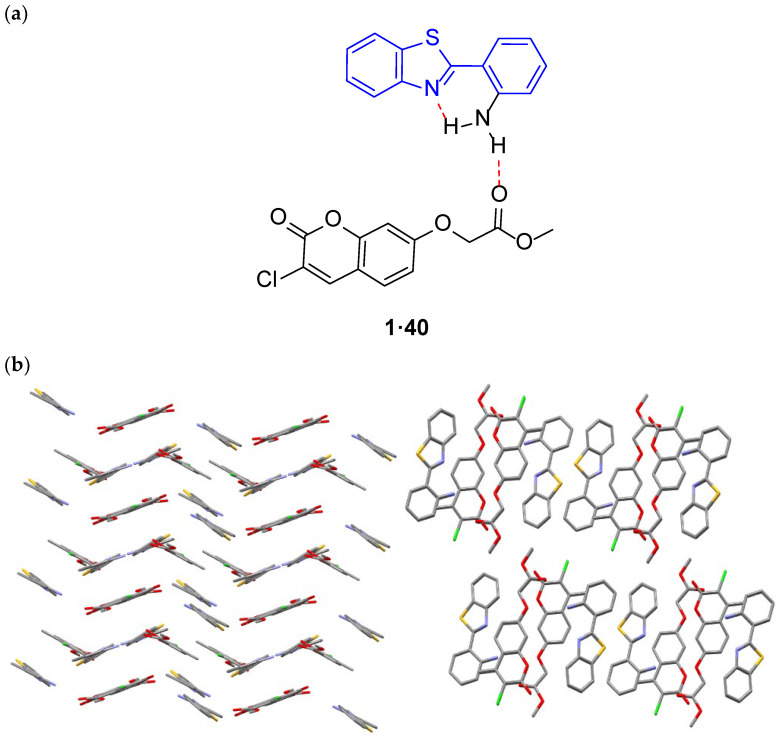
(**a**) Chemical structure of cocrystal of **1** with methyl 2-((3-chloro-4-methyl-2-oxo-2H-chromen-7-yl)oxy)acetate **40**, involving hydrogen bonds; (**b**) X-ray structure of known cocrystal **1·40** [116].

Compound **1** is an aromatic amine and exhibits chemical properties characteristic of this class of compounds. For example, **1** formed cocrystals with planar aromatic systems, such as methyl 2-((3-chloro-4-methyl-2-oxo-2H-chromen-7-yl)oxy)acetate (**40**) (Figure 3), as it was presented by Al-Amiery et al. [116]. In these cocrystals **1·40**, two aromatic molecules formed zigzag chains via hydrogen bonds between the amino group of **1** and the keto-group of coumarin derivative, along with π-π-stacking interactions.

**Scheme 13:**
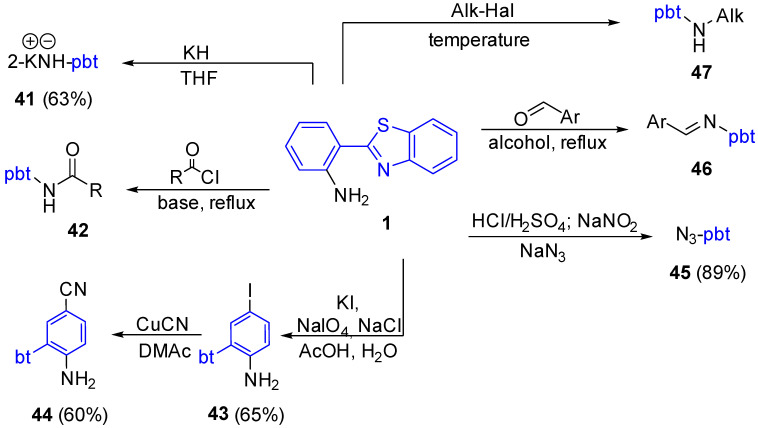
Overview of chemical transformations of **1**.

Deprotonation of **1** occurs readily from the reactions with strong bases in organic solvents, providing the corresponding amido alkali salts **41** (Scheme 13) [84,85]. The latter can be further introduced in nucleophilic substitution reactions to synthesize phosphorus and silylated derivatives (see below). Furthermore, the amino group in **1** can be modified by nucleophilic substitution reactions yielding amides **42** (Scheme 13). From a sensor development point of view, these compounds are especially interesting because their photophysical properties, along with chemical properties, allow for their use as selective probes [77,108,117,118,119]. Moreover, the electrophilic substitution of hydrogen in the phenyl ring of **1** to the corresponding iodo and further to the cyano derivatives can be performed in the reaction of **1** with KI in acid medium and then with CuCN in DMAc (Scheme 13, **43,** and **44**) [97]. Other transformations of the amino group to the azide **45** [84,91], imine **46** [27,120,121,122], and alkyl amine derivatives **47** can be readily achieved from **1** (Scheme 13) [75,92].

#### 2.2.4. Functionalization of 2-(2′-Aminophenyl)benzothiazole with Phosphorous Groups

**Scheme 14:**
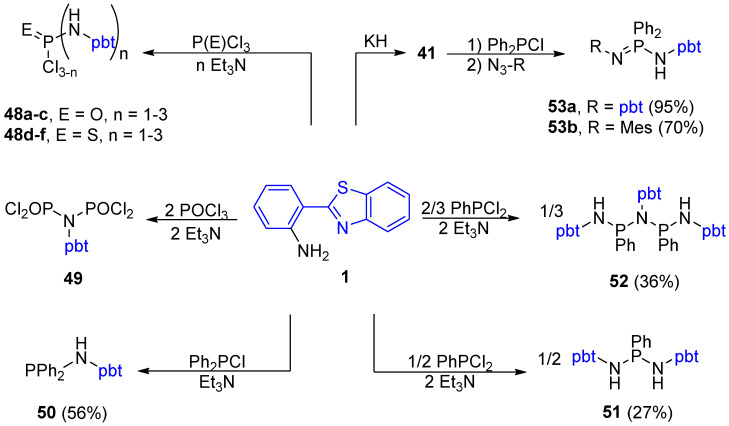
Synthesis of phosphorus derivatives of **1**.

The phosphorous chemistry of compound **1** has started to develop over the last two decades. The first examples were reported by Mittal et al. in 2008. They synthesized phosphorylated and oxo-/thiophosphorylated derivatives of **1** (Scheme 14, **48a**–**f** and **49**) and studied their antifungal activity. However, the SCXRD data were not given for any of these examples; all derivatives were characterized by IR-, NMR-spectroscopy, and mass spectrometry [41].

**Scheme 15:**
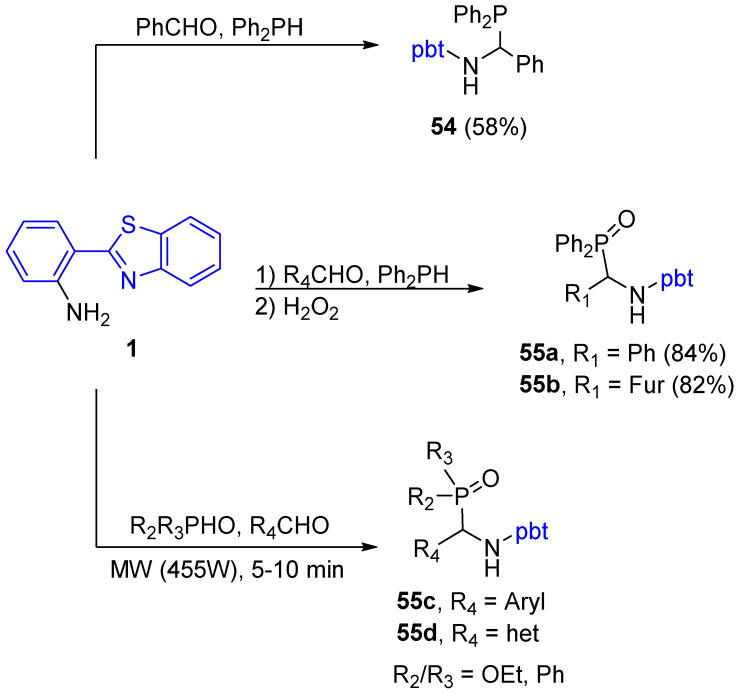
Synthesis of 1,3-aminophosphonate and 1,3-aminophosphine derivatives of **1**.

A significant contribution to the development of phosphorus derivatives of **1** has been made by the Konchenko group. Several new phosphorus-containing compounds have been synthesized using nucleophilic substitution reactions (Scheme 14, **50**–**52**) [82], Staudinger reactions (Scheme 15, **53a**–**b**) using potassium salt **41** [84,85] or phospha-Mannich reactions (Scheme 15, **54** and **55a**–**b**) [50,83]. α-Aminophosphonates (Scheme 15, **55c**–**d**) were obtained by Basha et al. in order to study their biological activity [103]. The chemical structures of these compounds are shown in Scheme 14 and Scheme 15.

#### 2.2.5. Functionalization of 2-(2′-Aminophenyl)benzothiazole with Silyl Group

**Scheme 16:**
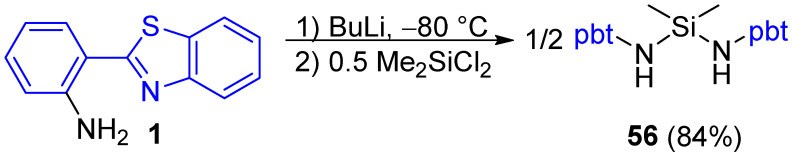
Synthesis of silanediamine framework **56** derived from **1** [48,131].

From a coordination chemistry perspective, another promising bidentate proligand is the silanediamine **56** (Scheme 16), which, in its doubly deprotonated form, can coordinate to one or two metal centers. Generally, silanediamide ligands are capable of stabilizing low-valent oxidation states as well as mixed-valence dimeric complexes M(I)/M(II) [123,124,125,126]. The conformational flexibility of the silanediamide ligand allows for the coordination of metals with large atomic radii [125,127,128,129,130]. The incorporation of a photophysically active core into the silanediamine framework could lead to remarkable properties. To explore this potential, Mironova et al. synthesized compound **56** derived from **1** [48,131]. They used a common approach [132,133]; the deprotonated amine reacted with dimethyldichlorosilane in a nucleophilic substitution reaction, providing **56** in an 84% yield.

Overall, the mentioned compounds are also promising as pro-/ligands for coordination chemistry due to the presence of phosphorus, nitrogen, and, in some cases, chalcogen (O or S) or silicon atoms, which could lead to intriguing chemical reactivity [50,131] or formation of interesting coordination architectures with great potential in luminescent applications, as discussed below.

### 2.3. Luminescence Properties: ESIPT, AIE

Compared to other organic chromophores, the most intriguing properties of derivatives of **1** are characteristic Excited-State Intramolecular Proton Transfer (ESIPT, Section 2.3.1.) and Aggregation-Induced Emission processes (AIE, Section 2.3.2.). These processes, which are mainly observed in substituted amino derivatives (2-RNH-pbt) bearing electron-withdrawing groups, will be discussed in detail below.

#### 2.3.1. ESIPT

**Figure 4:**
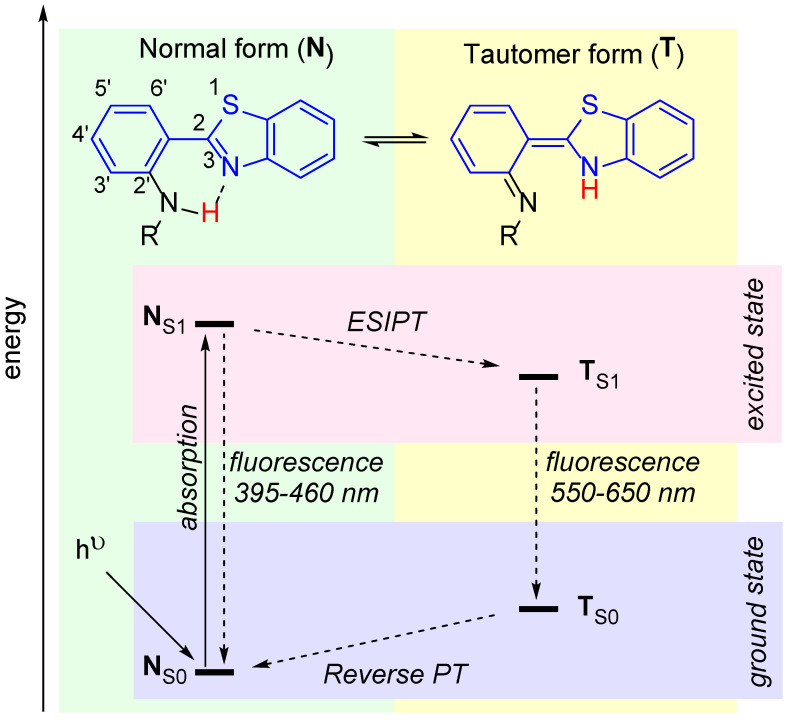
ESIPT process in 2-RNH-pbt derivatives [134].

This photochemical process occurs due to the difference in proton acidity between the ground state and the excited state (Figure 4). 

The photochemical cycle involves four states: excitation of the ground state (N_S0_) leads to the formation of the normal form of the excited state (N_S1_), followed by ESIPT, resulting in the tautomer excited state (T_S1_). Fluorescence then occurs, forming the tautomer ground state (T_S0_), and reverse proton transfer (RPT) takes place. The energy of the transition from the excited state of the tautomer (T_S1_) to the ground state of the tautomer (T_S0_) is smaller than that of the normal form (i.e., N_S1_→N_S0_); for this reason, the ESIPT band appears as a red-shifted band in the spectrum. Due to the ultrafast rate of ESIPT (<<1 ps), studying this process exceeds the capabilities of most modern detection systems. This remarkable process is often displayed in various OH-type organic compounds [134,135,136], among which 2-(2′-hydroxyphenyl)benzothiazole derivatives [137,138,139,140,141,142,143] play an important role. Intramolecular hydrogen bonds in OH-type frameworks are generally stronger than those in NH-type systems. This increased hydrogen bond stability facilitates ESIPT in the former compounds. In contrast, in NH-type compounds, the energy barrier for ESIPT is higher [144]. However, the advantage of the 2-RNH-pbt framework over 2-(2′-hydroxyphenyl)benzothiazoles lies in its tunability: the introduction of donor/acceptor substituents both in the phenyl ring and amino group can influence the HOMO-LUMO gap. The ESIPT process was clearly demonstrated in aminopbt derivatives by Tseng et al. [97]. They studied a series of compounds (Table 6, **1**–**2**, **15**, **44**, and **57**–**60**) and showed that the possibility of ESIPT increases in the following order: **2** < **1** < **57** < **15**. The strong acceptor substituent (i.e., Tos) at the amino group accelerates ESIPT, while a donor substituent (i.e., Me) prohibits this process. Ultrafast ESIPT was observed in **15**, there is only emission of the tautomer form of **15** with a large Stokes shift (Table 6). While the derivative with a less electron-withdrawing group **57** exhibited a slower process, there were two bands corresponding to fluorescence from N_S1_ and T_S1_ states (i.e., normal and tautomer, Table 6).

**Figure 5:**
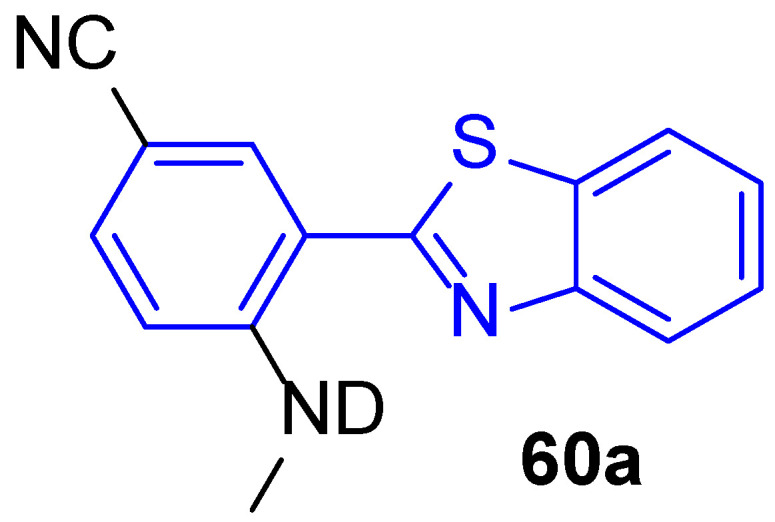
Deuterated derivative of **60a** [97].

Examples of tuning through the introduction of donor/acceptor groups in the phenyl ring include compounds bearing NH_2_- and CN-groups in para-position to the tosyl-substituted amino group in **15**: 2-(2′-(N-tosyl)amino-5′-aminophenyl)benzothiazole (Table 6, **58**) and 2-(2′-(N-tosyl)amino-5′-cyanophenyl)benzothiazole (Table 6, **59**). Even though all compounds in this series exhibit only the ESIPT process and do not demonstrate fluorescence from the N_S1_ state, the introduction of substituents can tune the maximum of emission: emission bands are shifted towards the red region, in the order **59**→**15**→**58** (540 nm→555 nm→649 nm). In contrast, the introduction of a CN substituent in the 5′-position 2-(2′-(N-methyl)amino-5′-cyanophenyl)benzothiazole (Table 6, **60**) leads to a weak ESIPT process, while **2** shows only the strictly non-proton transfer spectra. Moreover, a combined approach including kinetics, spectroscopy data and theoretical calculations was applied for the determination of the ESIPT mechanism. The kinetic experiments with non-deuterated **60a** (Table 6) and deuterated **60a** (Figure 5) showed identical resolved kinetic traces, indicating the absence of a kinetic isotope effect. The authors suggested that the rate-limiting step of ESIPT in aminopbt derivatives would be influenced by a change in the molecular core, with N^2′^ and N^3^ moving closer to each other to transfer a proton rather than by the proton transfer itself [146]. As a result of the research, the modification of the **1** core can control the rate and energy barrier of the ESIPT, as well as photophysical properties [97].

As it was shown, the introduction of an amide group at the 2′-position in ***pbt*** derivatives facilitates the ESIPT process, as observed in **15** and **57**. This behavior was also demonstrated in benzoyl-amides (Table 6), which turned out to be promising compounds for OLED technology. Bright white-light emission with high fluorescence quantum yields (Φf = 23–35%) was observed in **30e**, **30f**, and **30g** in the solid state (Table 6). All three compounds exhibited two-band emission in the spectra [62], while the **3** (Table 6) did not undergo the tautomer form at the excitation neither in solid state nor in solution (CHCl_3_) [76], as confirmed by the theoretical study [147]. In contrast, Tseng et al. observed a slow ESIPT in **44** (Table 6), where the acceptor group (i.e., CN) was placed in 5′-position. Thus, it could be concluded that only the introduction of acceptor groups in the 5′-position of the phenyl ring and at the amino group can lead to the appearance of a second band (ESIPT-band) in the spectrum.

Tseng’s study attracted significant attention for the investigation of ESIPT-active derivatives of **1** using DFT calculations. Most of the calculations were performed with the TD-DFT method in Gaussian 09 program package. The first theoretical research works demonstrated that ESIPT-active compounds can undergo isomerization around the C^2^-C^1′^ bond in the excited state (Table 7). This twisting brings the S_1_ and T_1_ potential energy curves close together, enabling their intersection. As a result, ISC occurs from T_s1_, populating both T_T1_ and twisted-T_T1_. Then, these states relax via nonradiative decay to form T_s0_ and twisted-T_s0_. The authors calculated that twisted-T_s0_ must overcome a significant energy barrier to return to N_s0_ form. Based on their data, they concluded that ESIPT-active compounds in the ground state may exist as long-lived twisted-T_s0_ species [148,149]. Moreover, another study found that a higher free energy of the ESIPT reaction correlates with a higher reaction rate [136]. Calculations of ESIPT energy barriers for **1** and **2** suggest that the methyl donor group hinders the ESIPT process, making it less favorable [148,149,150]. Obviously, the ESIPT in derivatives of **1** can be tuned by introducing donor or acceptor groups at the amino group or 5′-position. However, the effect of the substituent does not always directly correlate with the strength of the intramolecular hydrogen bond, which means the prediction of the ESIPT trends based on only substituent effects may lead to an incorrect effect [136].

Singh et al. studied the luminescent properties of a set of ***pbt*** derivatives (Table 8). A drastic difference in absorption spectra was observed for **33m**, **33o,** and **33p**, whereas for other compounds, the absorption maximum remained around 380 nm. In fact, this study provides an intriguing confirmation of the lack of a correlation between substituents in ***pbt*** and the occurrence of ESIPT. In contrast to compound **44**, the authors did not observe the dual-band emission of compounds with F- and Cl-substituents at the 5′-position and donor substituents at ***bt*** heterocycle (**33g**–**33i** and **33l**) in the range of 250–525 nm in chloroform solution. Interestingly, the introduction of any substituent (whether a donor or acceptor group) at the 5′-position did not result in an emission shift for compounds **33g** (440 nm), **33h** (440 nm), and **33k** (440 nm), while the emission of parent **33j** is blue-shifted (425 nm) compared to its substituted derivatives (Table 8). Conversely, replacing the methoxy group with F- or Cl-substituents in **33o**, **33i**, and **33l** leads to a hypochromic shift in the emission spectra. The introduction of two donor groups into the ***bt*** core led to a red-shifted emission for **33m**-**33o** compared to **33p** (Table 8, 470 nm and 465 nm) [114].

**Figure 6:**
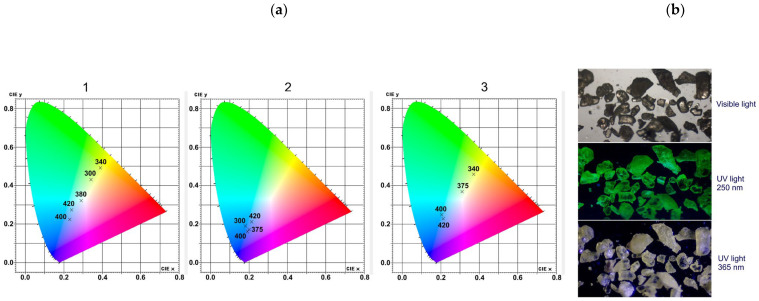
(**a**) Chromacity CIE x,y coordinates of **50** (1), **51** (2), and **52** (3) showing emission colors upon different excitation wavelengths, (**b**) photographs of crystals on the example of **50** under visible transmitted light, UV light with the maximum at 250 nm and UV light with the maximum at 365 nm, adapted from ref. [82].

Functionalization of **1** with diphenylphosphine further modifies the luminescent properties of benzothiazoles (Table 9). Specifically, all reported compounds in the solid state demonstrate dual-band emission due to the occurrence of both normal (N_S1_) and ESIPT-induced (T_T1_) excited states. In addition, **50**, **51,** and **52** reveal the possibility of twisted-T_S1_ as well. According to TD-DFT calculations, the excited-state twist in the compounds occurs around the C–N(H) bond, resulting in the appearance of one more excited state capable of radiation decay. Changing the excitation wavelength provides the possibility to change the relative intensity of the two emission bands, which results in a glowing color change from green through white to blue (Figure 6). The observed lifetime of the excited state lies in the range of 30–700 μs, implying a phosphorescent nature of the emission, which is especially important for high-performance OLED devices. The emission of α-aminophosphonates (**55a** and **55b**) and iminophosphonamine **53a** is bathochromically shifted compared to the P(III) derivative **54**. The combination of the transitions from the excited states enables tunable emission depending on the excitation wavelength [82,84].

#### 2.3.2. AIE

**Figure 7:**
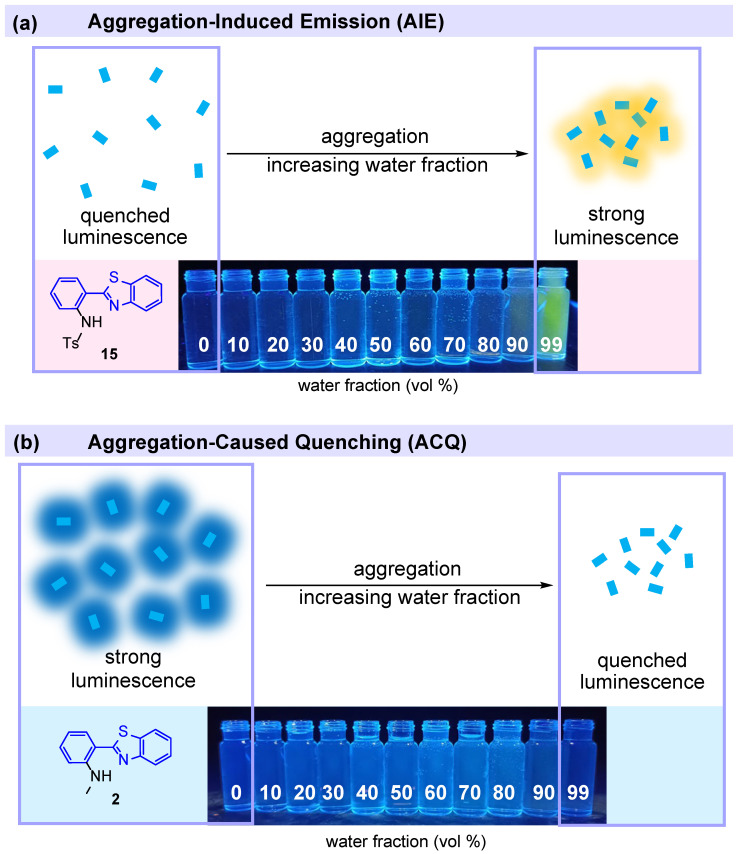
(**a**) AIE and (**b**) ACQ phenomena of 2-aminopbt derivatives [151], adapted with permission from reference [98], Copyright@Elsevier 2023.

Not only is ESIPT characteristic of fluorophores based on ***pbt*** derivatives, but the 2′-amino-pbt compounds can also have Aggregation-Induced Emission (AIE) properties [98]. AIE is a complex and not fully understood phenomenon, which has been reported in conjugation with different additional photophysical processes, and a complete mechanism of AIE has not been described yet. The compounds exhibiting AIE emit light more intensively by aggregation, which typically occurs when water fractions are added to their organic solutions (Figure 7a) [151]. Intra- and intermolecular interactions, such as π–π, dipole–dipole, hydrogen bonding, halogen bonding, and others, are known to affect the AIE behavior [152]. ***Pbt*** derivatives characterized by ESIPT (i.e., those with electron-withdrawing groups at 2′-amino group) tend to demonstrate AIE behavior, which is related to the twisted intramolecular charge transfer (TICT) process. In contrast, the derivatives with electron-donating groups at amino group in the 2′-position, where ESIPT does not occur, exhibit aggregation-caused quenching (ACQ) behavior (Figure 7b) [98].

**Figure 8:**
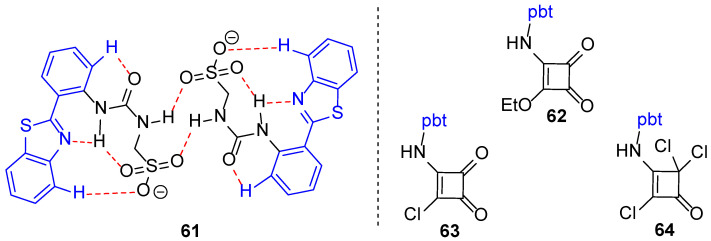
2-RNH-pbt frameworks showing AIE properties [78,153].

Hydrogen bonding is one of the frequently encountered examples that promote AIE. Several types of hydrogen bonds (Figure 8) were found in the tetrabutylammonium sulfonate–urea amphiphilic salt **61** derived from **1**, which plays an important role in the formation of self-associated dimers [78]. Another example combining ESIPT and AIE in ***pbt*** derivatives was discussed by Liang et al. They synthesized a series of 2-RNH-pbt-based semisquaraines with different numbers of electron-deficient chloro-substituents in a four-membered ring (Figure 8, **62**–**64**). The greater number of chloro-substituents in the ring promotes easier ESIPT, and the structure has a more distorted configuration. This is an example of the AIE phenomenon, which occurs due to extremely weak π-π interactions in the solid state, enhanced by multiple halogen bonds [153].

#### 2.3.3. Miscellaneous Photophysical Properties

Another way of tuning the emission band of **1** is via the synthesis of organic nanoparticles (Lewis-pair nanoparticles). Considering the amine as a Lewis base, the combination of **1** with another Lewis acid (diphenylborinic anhydride) leads to the formation of nano-Lewis-pair adducts, which have already exhibited dual-emission. The variation in chromophore concentration during the step of nanoparticle synthesis results in different fluorescence colors of products, going from light-blue, yellowish-pink, to orange [154].

**Figure 9:**
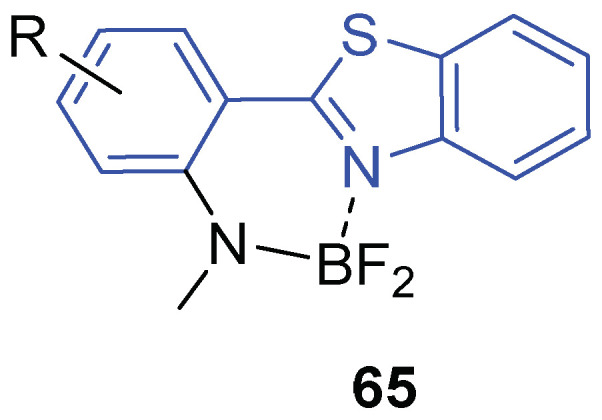
2-MeNH-pbt derivatives **78** bearing a boron difluoride moiety [155].

Interesting results were obtained by Tseng et al., who studied boron complexation of 4′/5′-substituted 2-MeNH-pbt derivatives with BF_3_·Et_2_O (Figure 9, **65**). In the synthesized complexes, the ligand had a nearly planar structure, which reduced the non-radiative decay channel associated with structural relaxation. These complexes exhibit higher quantum yields than the starting amines, and their emission changes from green, yellow, to orange depending on the substituents on the 4′/5′-position [155].

**Figure 10:**
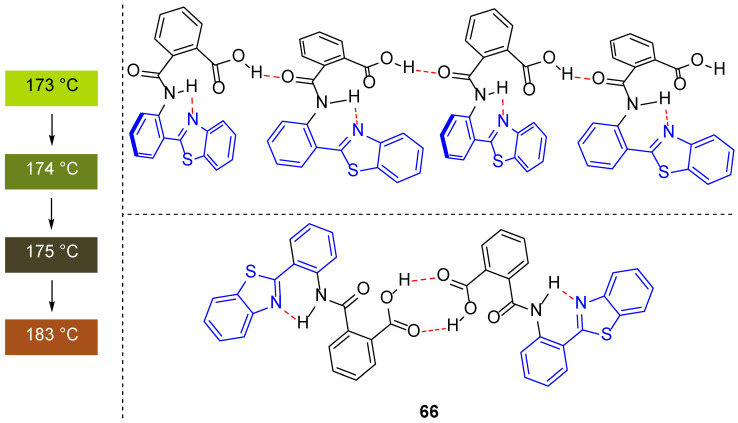
Molecular structures of two crystalline modifications of 2-aminopbt derivative **79** (top and bottom) and change in the glow of a single crystal with an increase in temperature under UV irradiation (left) [81].

Another intriguing study was performed by Kuz’mina et al. They carried out the condensation of **1** with phthalic anhydride and isolated two different crystalline forms and one amorphous form of the synthesized product **66** (Figure 10, top and bottom). Phase transitions were found between the three forms: the local melting of the crystal led, simultaneously, to the crystallization of another form with a higher melting point (Figure 10, left) [81].

Thus, the various 2-amino-pbt derivatives demonstrate remarkable luminescent responses toward changes in their geometry and\or intermolecular contacts. This intriguing feature is generally studied in terms of AIE or ACQ, arising upon transition from a solution to a solid state. Specific mechanisms for AIE or ACQ are still not straightforward, and the appearance of these phenomena in a certain compound is hardly predictable. However, when designing pbt compounds and interpreting their luminescent properties one may consider the following possibilities: (1) hydrogen bonding, responsible for ESIPT, which is generally more pronounced in the case of derivatives with acceptor substituents in 5′-position of the phenyl ring and at the amino group; (2) formation of π-π interactions upon aggregation in compounds with extended conjugate π system also may be responsible for AIE or ACQ; (3) different TICT processes may become more prominent for derivatives with non-rigid moieties, giving rise to low-lying excited states capable of radiation decay.

## 3. Overview of the Coordination Chemistry of 2-(2′-Aminophenyl)benzothiazole Derivatives

The coordination chemistry of **1** and its derivatives is still in its early stages, even though it offers a promising way to synthesize highly functional compounds. The mutual influence of metal ions and ligands bearing a photoactive core can enhance the luminescent properties and lead to new attractive effects, especially also for biological applications. However, only several examples of characterized complexes are known, including studies on the coordination compounds of Co(II) [156], Ni(II) [157,158], Zn(II) [145], Re(I) [159,160], Re(V) [161] with **1** as a ligand. A few Pt(II) and Pd(II) complexes of various derivatives of **1** have been synthesized, and a series of coordination compounds of rare-earth metals with silanediamine [48,131] and iminophosphonamide [84,85] derivatives of **1** were recently reported.

### 3.1. Coordination Complexes with Neutral Ligands Based on 2-(2′-Aminophenyl)benzothiazole

**Figure 11:**
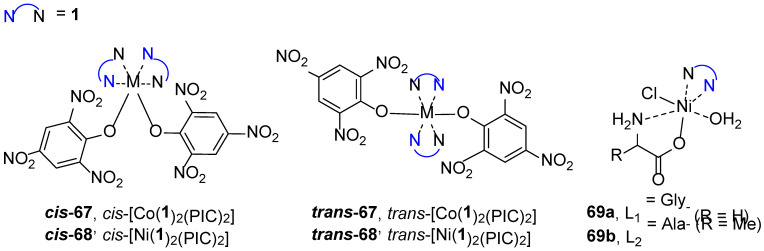
Proposed chemical structures of the Co and Ni coordination compounds with neutral **1** [156,157,158].

The first examples of metal–organic compounds with the parent ligand **1** were the picrate complexes [Co(**1**)_2_(PIC)_2_] (Figure 11, **67**) [156] and [Ni(**1**)_2_(PIC)_2_] (PIC = (NO_2_)_3_C_6_H_2_–O^−^) (Figure 11, **68**) [157], for which the authors proposed two possible *cis*- and *trans*-isomers. None of the known Co(II) and Ni(II) complexes have been crystallized, so their structures were confirmed by combined physicochemical methods. In all cases, **1** coordinates in a chelate manner via the N^3^-N^2′^ atoms. Another heteroligand [Ni(**1**)(H_2_O)(L)] (Figure 11, **69a**—L_1_ = Gly^−^, **69a**—L_2_ = Ala^−^) complex has been studied for application against pathogenic fungi, such as *Aspergillus niger* and *Fusarium oxysporum* [158].

**Figure 12:**
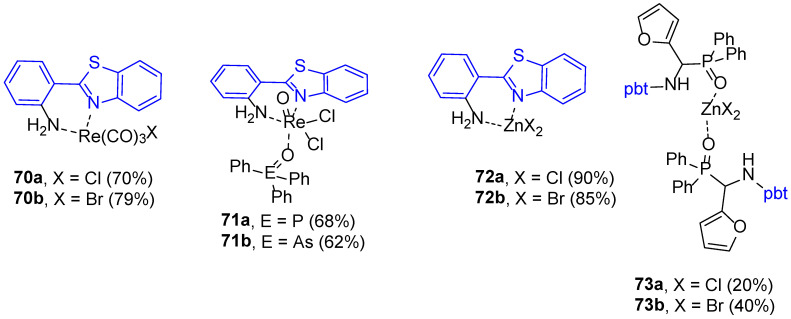
Structurally characterized Re and Zn coordination compounds of **1** and comparison with phosphine oxide complexes **86** [145,159,160,161].

Later, two tricarbonyl complexes of Re(I) with **1** (Figure 12, **70a** and **70b**) were synthesized [159,160]. The luminescent properties of *fac*-**70a** were investigated. The absorption spectra showed transitions characterized by LLCT and IL transitions involving **1**. DFT calculations were used to correlate the transitions in both absorption and emission spectra. The obtained data allowed the authors to suggest potential applications in optoelectronic devices [160].

Two more metal–organic compounds with parent **1** have been reported, both of which are oxorhenium(V) complexes (Figure 12, **71a** and **71b**). In both complexes, the central Re(V) atom is placed in a six-coordination sphere with distorted octahedral geometries, where **1** and two chloro ligands occupy the equatorial plane. The absorption spectra revealed bands in the range of 300–450 nm, which were assigned to metal-to-ligand charge transfer (MLCT) transitions involving **1**, while highly intense absorption bands in the range of 250–300 nm originate from LMCT and LLCT transitions [161].

Recently, two zinc halide complexes with **1** were published [145]. Even though the reactions were carried out in the same way—reaction in ethyl acetate with a zinc halide (ZnCl_2_ or ZnBr_2_) to **1** ratio of 1:1 (Figure 12, **72a** and **72b**)—two different structures were defined via SCXRD. Compound **1** coordinates to zinc in a chelating manner, but the organic ligand in the chloro complex **72a** exhibits a planar structure, whereas, in the bromo **72b** complex, the planes of the benzothiazole fragment and the phenyl ring are positioned at an angle of 23–31° relative to each other. Despite the conjugation between the heterocyclic fragment and the phenyl ring in the ligand, **1** demonstrates the flexibility of the organic backbone around the C^1′^-C^2^ bond (Table 1). The reason for the structural difference remains unclear. Authors compared the mentioned complexes with a similar series of halide complexes with **55b** ligand (Figure 12, **73a** and **73b**). The oxidized phosphine ligand coordinated with the zinc center exclusively via the oxygen of the P=O moiety, while the heterocycles did not participate in the coordination. In all complexes, Zn(II) is in a tetrahedral environment. The ***pbt*** core in the ligand is not plane in both **73a** and **73b** (X = Cl (3.6◦), Br (15.0◦)) with smaller angles between the heterocycle and aniline moiety than in bromo complex **72b**. The photoluminescence properties were studied for these complexes. The emission spectra corresponding to the **72a** and **72b** complexes exhibit a hypsochromic shift in the emission maxima compared to the free ligand; a redshift is also observed as the ligand transitions from chloride to bromide. In contrast to amine complexes, **73a** and **73b** exhibit two-band emission with maxima at 450 and 600 nm. The authors assumed the first band corresponded to a radiative transition for the regular amine species, while the second band can be explained either with the ESIPT or TICT.

**Figure 13:**
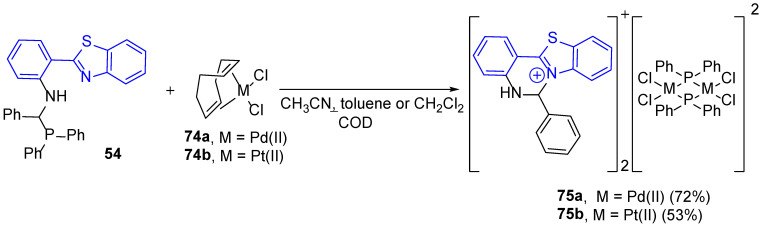
Activation/cyclization reactions of the P-C bond in reaction of **54** with **74a** or **74b** [50].

In work carried out by Afonin et al., the reactivity of [M(COD)Cl_2_] (M=Pd(II), Pt(II), COD = cycloocta-1,5-diene) with ligand **54** ligand derived from **1** was investigated. It was found that reactions between Pd(II) and Pt(II) complexes (**74a** and **74b**) with **54** resulted in the activation/cyclization reactions of the P-C bond, and further P−C bond cleavage led to the formation of PPh_2_^−^ species. This rearrangement of **54** afforded the formation of a heterocyclic fragment, representing a cation with the cycle {N-C-N-C-C-C} (Figure 13, **75a** and **75b**). Consequently, the ligand could not be introduced into the inner coordination sphere of the metals. A mechanism for the P-C bond activation/cyclization was suggested using quantum chemical calculations [50].

### 3.2. Coordination Complexes with Anionic Ligands Based on 2-(2′-Aminophenyl)benzothiazole

**Figure 14:**
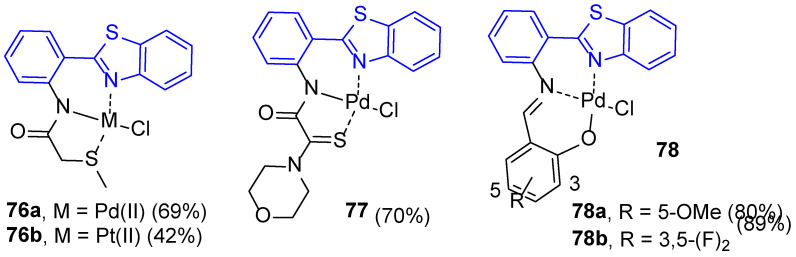
Structurally characterized metal–organic compounds of Pd- and Pt-based on S,N,N and O,N,N pincer derivatives of **1** [72,162,163].

Other biologically active complexes were synthesized with a methylthioacetamide ligand attached to **1**. The Pd(II) and Pt(II) complexes with this ligand (Figure 14, **76a** and **76b**) were obtained in moderate yields and structurally [162]. Consequently, the discovery of an alternative method for synthesizing a palladium complex with an S,N,N-pincer ligand was reported (Figure 14, **77**). This method employed a mechanochemical approach that yielded the complex quantitatively within less than 7 min by grinding in a vibrational ball mill, compared to up to 2 days for solution synthesis [163]. These complexes **76** and **77** were designed to enhance binding with biological targets through stacking interactions. Moreover, cytotoxicity tests were performed on various human cancer cell lines (human colon, breast, and prostate cancer), yielding promising results. The study showed that the coordination to Pd(II) or Pt(II) was crucial for the complexes to function as effective anticancer agents [162,163].

Due to the known effectiveness of palladium complexes against tumor cells, a series of Pd(II) metal–organic compounds with salicylaldimine-based benzothiazole derivatives with different substituents in phenyl ring were synthesized, and their cytotoxicity was studied (Figure 14, **78**). The interaction of each complex with circulating tumor DNA was studied in a combined approach involving absorption spectroscopy, competitive fluorescence quenching assays, and molecular docking simulations. The complex with the 5-methoxysubstituted ligand (**78a**) exhibited the best cytotoxicity against the liver cancer cells, while the complex with the 3,5-difluorosubstituted ligand (**78b**) demonstrated the highest anticancer activity against the breast cancer cells. These findings highlight the potential of designing **1** derivatives, allowing flexible cytotoxicity tuning of the corresponding Pd(II) complexes [72].

**Figure 15:**
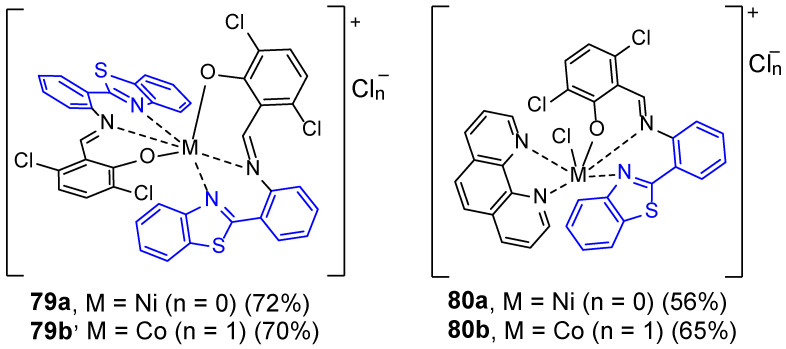
Proposed structure of metal–organic compounds of Ni(II) and Co(III) based on salicylaldimine-based derivatives of **1** [51].

Similarly to **78a** and **78b** complexes (Figure 15), bis-ligand and hetero-ligand Ni(II) (**79a** and **80a**) and Co(III) (**79b** and **80b**) complexes were synthesized with ligand based on 2,5-dichlorosalicylaldehyde and **1**. The structures of these complexes were confirmed through a combination of physicochemical methods (elemental analysis, electronic absorption, mass and NMR spectral studies, and thermal analysis). The research suggests the use of mixed ligand complexes with 1,10-phenanthroline as antibacterial agents against both *Grampositive* and *Gram-negative bacterial strains*. Moreover, at increasing concentrations of mixed ligand complexes, the number of cancerous lung tissue cells decreased in vitro [51].

In contrast to zinc complexes with neutral **1** (Figure 12, **72a** and **72b**) [145], it turned out that two deprotonated [2-RN-pbt]^−^ ligands can bind in the inner coordination sphere of Zn(II). For this, zinc chloride was treated with the sodium salts of the ligands (NaL**^81−85^** and NaL**^15^**) in a 1:2 ratio. These compounds were used for the preparation of the OLED devices with the blue color of the resulting diode (Table 10) [164,165,166,167]. The authors used the new complexes as emission layers or electron-transport layers with various hole-transport layers (i.e., PTA, NPD, or CBP). The designed cells showed good brightness at low voltage.

**Figure 16:**
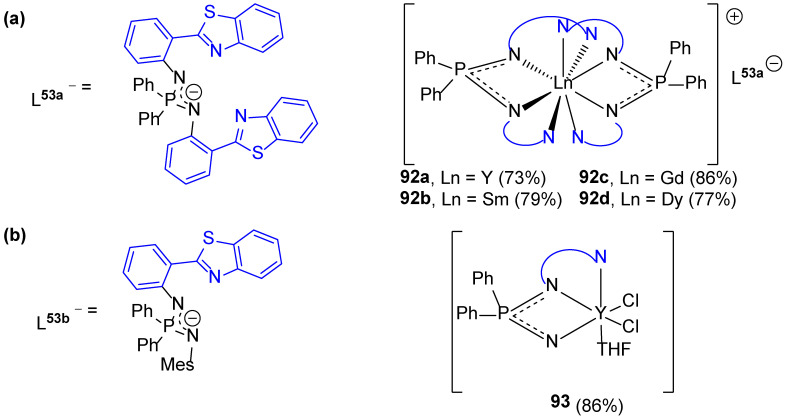
Structurally characterized rare-earth metal complexes with (**a**) anionic ‘symmetrical’ NPN derivative of **1** and (**b**) anionic ‘non-symmetrical’ NPN derivative of **1** [84,85].

Two studies were performed on the luminescent properties of trivalent rare earth (Ln) complexes with an iminophosphonamide ligand (L**^53a^**^−^ and L**^53b^**^−^) by Sinitsa et al. (Figure 16a,b). The reaction of **53** with potassium hydride in THF led to the formation of potassium salt of iminophosphonamide (L**^53a−^** and L**^53b−^**) and addition of lanthanoid halides in stochiometric amounts afforded complexes **92** (ratio LnCl_3_: L**^53a^**^−^ = 1:3) and **93** (ratio YCl_3_: L**^53b−^** = 1:1). Emission spectra with narrow bands were obtained for the samarium(III) coordination compound with ‘symmetrical’ (i.e., having same substituents at the N atoms) L**^53a−^** ligand, indicating a low-symmetry coordination environment and that the iminophosphonamide ligand exhibits an antenna effect. In the series of lanthanoid complexes with the ‘symmetrical’ iminophosphonamide ligand (Figure 16, **92a**–**d**), it was demonstrated that both the ligand and the lanthanoid atom influence the emission behavior, with the emission color ranging from turquoise to orange depending on the metal [84]. In contrast to known synthetic methods for silanediamines, it is possible to obtain ‘non-symmetrical’ iminophosphonamines, which can be used for studying the structural features of coordination compounds. Thus, **53b** based on **1** and mesitylaniline (NH_2_-Mes) and its yttrium complex **93** were synthesized (Figure 16) [85]. Due to the presence of chloro ligands in the inner sphere of the yttrium complex, it is a suitable starting reagent for the design of various heteroligand complexes in the future.

### 3.3. Coordination Complexes with Doubly Deprotonated NSiN Ligands Based on 2-(2′-Aminophenyl)benzothiazole

**Figure 17:**
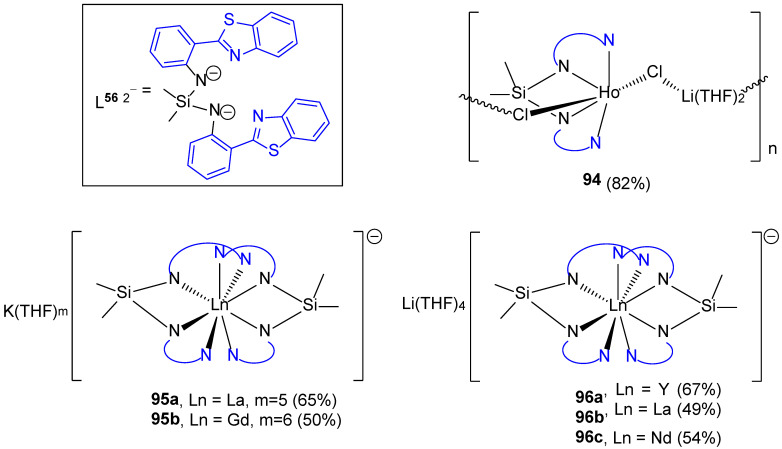
Structurally characterized rare-earth complexes with silanediamide ligand based on **1** [48].

The design of pincer ligands by introducing two **1** fragments enables the polydentate metal coordination, which is particularly suitable for larger ions, such as lanthanoids. The coordination via four nitrogen atoms provides the stabilization of rare-earth metals (Ln(III)) complexes in both solid state and solution. Additionally, the synthesis of polydentate ligands bearing fluorophoric groups aims to produce bis-ligand complexes [LnL_2_]^q^ (where q represents a charge of lanthanoid inner sphere). The increased rigidity of the deprotonated proligand in these complexes is beneficial for minimizing non-radiative luminescence decay. Thus, silanediamide ligand (L**^56^** ^2−^) was synthesized and introduced into a series of complexes with Y(III) (**96a**), La (III) (**95a** and **96b**), Nd(III) (**96c**), Gd(III) (**95b**), and Ho(III) (**94**) (Figure 17). **56** was initially deprotonated with ^n^BuLi or KBz in THF at −90 °C; then, lanthanoid chlorides reacted with the alkali salt. As a result, the smaller Ho(III) formed a polymeric complex **97** with two bridged chloro ligands, while the larger cations (such as Y(III), La(III), Nd(III), Gd(III)) coordinated two L**^56^** ^2−^ (Figure 17). In these bis-ligand complexes, two ligands interacted with each other via π-π interactions because of a helical arrangement of flat ***pbt*** fragments.

**Scheme 17:**
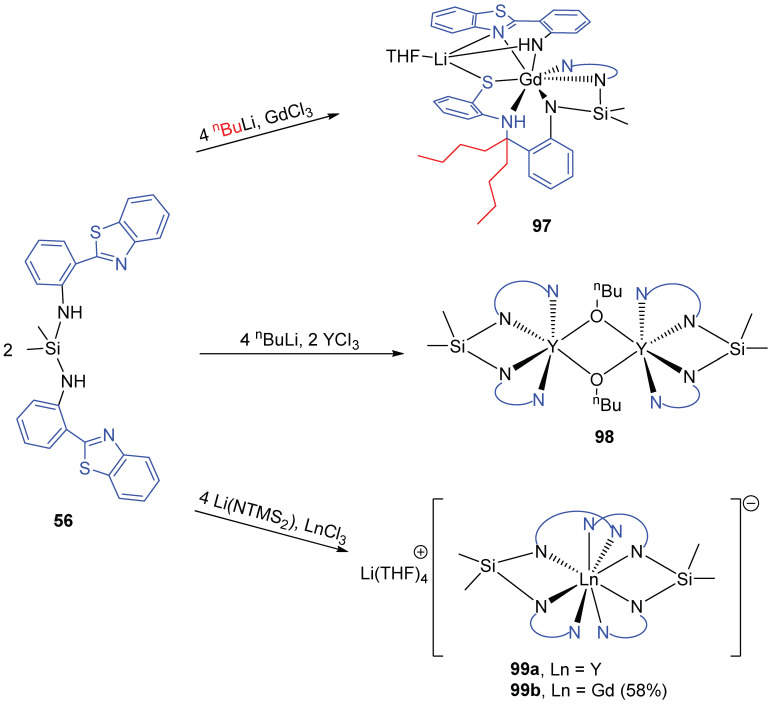
Unexpected formation of rare-earth complexes with silanediamide ligand L**^56^** ^2−^ [131].

However, in the case of Gd(III), the use of ^n^BuLi for deprotonation led to the unexpected formation of product **97** (Scheme 17), as the benzothiazole underwent nucleophilic addition of the n-butyl group [131]. To confirm that this was due to a local excess of ^n^BuLi in the reaction mixture, the same reaction with YCl_3_ was performed. NMR data supported the formation of a similar yttrium complex, although crystallization was unsuccessful. In the reaction of **56**, YCl_3_ and ^n^BuLi in a 1:1:2 ratio, **98** yttrium complex with bridged µ-O^n^Bu was isolated in small quantities, which was attributed to the presence of a LiO^n^Bu impurity in the starting material. Transitioning from ^n^BuLi to Li(NTMS_2_) in the reaction yielded the formation of the desired complex **99a** or **99b**.

The photoluminescent properties of the obtained compounds were studied, and although the estimated energy of triplet level was suitable for sensitizing the luminescence of Nd^3+^ and Ho^3+^ cations in the NIR region, no f-f bands were observed, except a weak band for holmium complex **97** [48]. Additionally, the triplet level of the silanediamide ligand L**^56^** ^2−^ was estimated using the Gaussian fitting of the emission spectrum of **95b** (16,905 cm^−1^) and **99b** (16,600 cm^−1^), which were recorded at 77 K [48,131].

In short, several transition-metal complexes with the neutral 2-amino-pbt ligand **1** are known, and their properties have been explored. Additionally, complexes with derivatives of **1** have also been studied, all of them bearing either deprotonated or doubly deprotonated ligands. In all cases, **1** and its derivatives typically coordinate in a chelate manner (i.e., N^3^ and N^2′^). An exception is the oxidized 1,3-aminophosphine **55b**, which coordinates to the Zn(II) center via oxygen atom (**73a** and **73b**). However, not all reactions lead to the expected complexes. For example, the 1,3-aminophosphine **54** can undergo rearrangement when reacting with Pd(II) or Pt(II), forming the ate complex **75a** and **75b**. Additionally, the benzothiazole heterocycle of silyldiamine **56** can react with strong bases, leading to nucleophilic addition. This makes the coordination chemistry of **1** and its derivatives an interesting area for further studies, including the use of other metal centers or the preparation of heterometallic compounds.

## 4. Overview of the Applications of 2-(2′-Aminophenyl)benzothiazole Derivatives

### 4.1. Applications as Small Molecule Sensors

**Figure 18:**
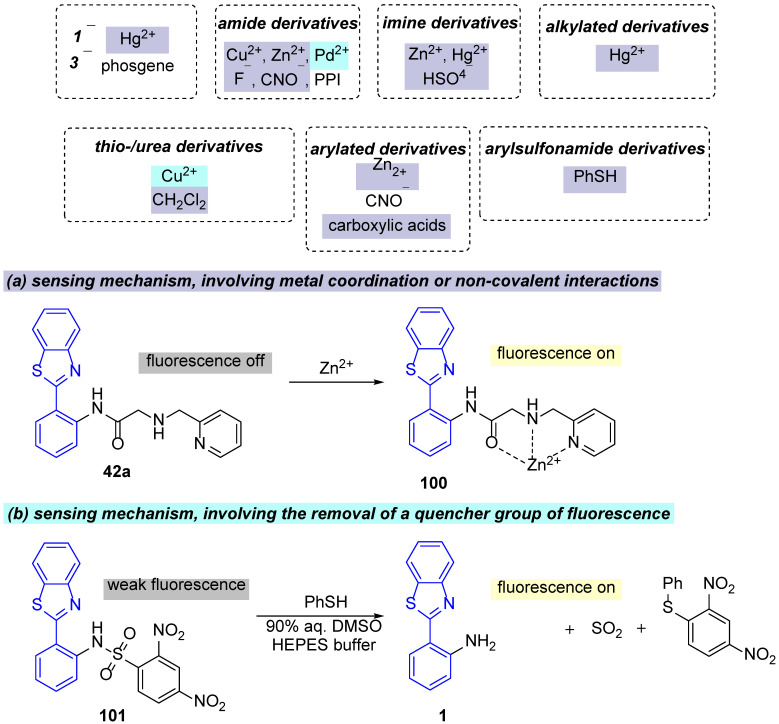
Overview of derivatives of **1** used for sensor applications and sensing mechanisms: (**a**) sensing mechanism, involving metal coordination or non-covalent interactions; (**b**) sensing mechanism, involving the removal of a quencher group of fluorescence [169,174].

Published studies on organic derivatives of **1** are mostly focused on the development of sensors for various ions (Figure 18). Extensive research on the detection of cations such as Fe^3+^ [27], Cu^2+^ [118,119,168], Zn^2+^ [77,169,170,171], Hg^2+^ [28,122], Pd^2+^ [108], as well as HSO_4_^−^ [27], pyrophosphate (PPI) [168], F^−^ [117], CNO^−^ [172,173], and cysteine [99], PhSH [174], carboxylic acids [175], CH_2_Cl_2_ [176], phosgene [177] clearly demonstrate these capabilities. Although the sensor mechanism for detecting transition metal cations is attributed to the coordination of the **1** derivative with the cation, no structures of coordination compounds have been described in these works. The mentioned sensors are typically amides of carboxylic acids, Schiff bases, or N-alkyl/aryl-substituted compounds. Two general mechanisms of sensor action are put forward. The first one (lavender box in Figure 18) involves the sensor acting as a polydentate ligand and coordinating through several additional donor atoms, leading to a change in luminescence intensity. In the case of anions, the strong non-covalent interactions between the sensor and sensing species lead to a luminescent response. The second mechanism (blue box in Figure 18) involves the removal of a fluorescence quencher in 2-RNH-pbts (see Figure 18b) or the cleavage of the C-N bond of the amide group (see Section 4.2.), which also leads to a luminescent signal change. For example, a dinitrobenzenesulfonate group quenches the luminescence of compound **101** due to its two strong electron-withdrawing groups (Figure 18b) [178]. In other cases, the sensing mechanism could involve various chemical reactions with sensing molecules [30,168,172,177].

### 4.2. Applications in APIs and in Biosensing

**Figure 19:**
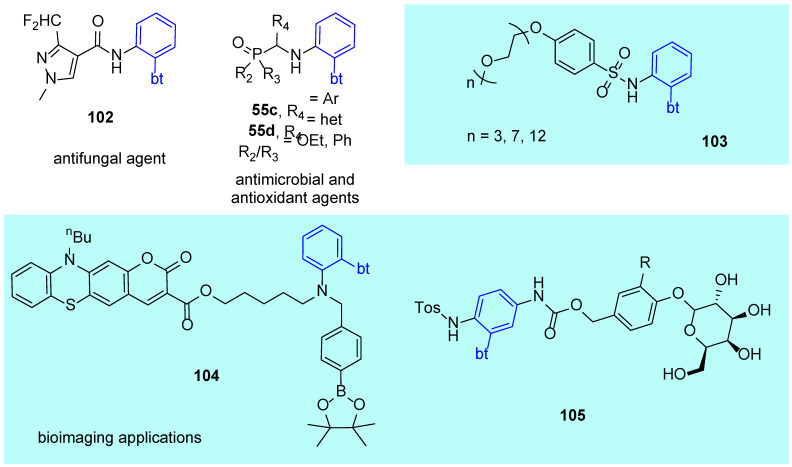
Some examples of compounds derived from **1** used in biological applications.

Several examples of biological applications of derivatives of **1** have been described (Figure 19). A number of articles show their significant potential for use as antifungal [107], antimicrobial, and antitumor agents and antioxidants [103,158], fluorescent probes [55,93,101,179,180,181,182,183] for bioimaging and sensors [99,100,121,176,184,185,186] in living cells.

In a comparative study [107], 21 compounds were evaluated against seven fungi (Colletotrichum orbiculare, Rhizoctonia solani, Phytophthora infestans (Mont.) De Bary, Pythium aphanidermatum, Fusarium moniliforme Sheld, Botryosphaeria berengeriana, and Botrytis cinerea) in vitro. Only one derivative of **1** was tested in this research. Difluorinated compound **102** (Figure 19) showed only moderate antifungal activity. In contrast, the compound bearing an indazole group instead of a **bt** fragment exhibited the highest antifungal activity, being, on average, 1.5 to 3.5 times more effective.

In a study by Thaslim Basha et al., compounds **55c** and **55d** (Figure 19) were tested for their antifungal (*Candida albicans, Candida non-albicans, Aspergillus niger*, *and Penicillium Chrysogenumby*) and antibacterial activity (*Bacillus subtilis*, *Staphylococcus aureus*, *Pseudomonas aeruginosa*, and *Escherichiacolib*) in vitro. Compounds **55d** exhibited the highest activity in both antifungal and antibacterial assays. Moreover, **55d** were the best antioxidant agents (especially those that bear the pyridinyl, thiophenyl, and imidazolyl substituents in the R_4_ position) [103].

Sensors **103** have been applied in the imaging of HeLa cells (Figure 19). Due to the ethyleneglycol tail, the developed compounds can enter the live cell membrane and form micelles, which significantly increases the intensity of its fluorescence [179].

Compound **104** was designed for separate or simultaneous detection of ClO^−^ and ONOO^−^ in the *Inflammatory RAW 264.7 Cells* and *Zebrafish* (Figure 19). The phenothiazine-based coumarin serves as a ClO^−^ sensor, and a boronate ester part of the **104** is responsible for tracking ONOO^−^. When detecting ClO^−^, the fluorescence color changes from red to green, while for ONOO^−^, it shifts from red to purple. If both anions are present, the color changes from red to blue. Due to high selectivity and wide applicability, this fluorescent probe was utilized in vivo and in vitro [180].

**Figure 20:**
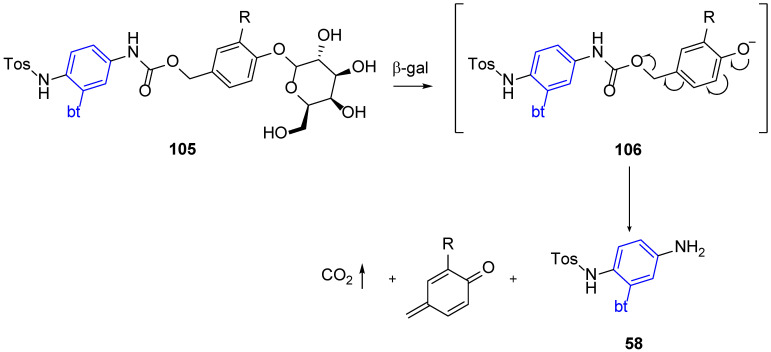
The mechanism of interaction of β-galactosidase with bio-probe **105** leading to a fluorescence signal change [101].

Two fluorescent probes **105** (Figure 19, R = H and NO_2_) were synthesized for β-galactosidase detection. These probes consist of ***pbt*** core linked via a self-immolative spacer to a hydrophilic galactopyranoside group, making them water-soluble. The β-galactosidase cleaves the chemical bond between galactopyranoside moiety and aromatic spacer, giving the intermediate **106** (Figure 20). Then, spacer self-elimination proceeded to yield a compound **58**. As discussed above, **58** is an ESIPT-active molecule (Table 6) with poor water solubility. Compound **58** exhibits an intensive NIR fluorescence signal upon AIE behavior (Figure 7a). Moreover, probes **105** have been successfully applied for long-term tracking in living cancer cells SCOV-3 (ovarian adenocarcinoma) and A549 (lung cancer) [101].

An extensive theoretical screening was performed by Kanlayakan and Kungwan, evaluating over 70 compounds as potential fluorescent probes for physiology, pharmacology, and molecular biology sensing in the visible region, including twenty 2-RNH-pbt derivatives. Authors establish several photophysical criteria for optimal ESIPT-based probes: (i) λ_abs_ should be close to the visible region (~380 nm); (ii) λ_em_ of tautomer form should range from 580 nm to 900 nm covering the NIR region; (iii) a large Stokes shift is essential to avoid self-reabsorption, which improves fluorescence efficiency of fluorescent probes; (iv) the kinetic and thermodynamic parameters, which provide the occurrence the ESIPT. Among the studied compounds, acetyl-containing derivatives met the first three criteria but exhibited a high proton transfer (PT) barrier, leading to a slow ESIPT. Additionally, the ESIPT in these molecules was endothermic in nature (Table 11). Moving from the acetyl group to a tosyl group lowered the PT barrier, making the ESIPT process more efficient and thermodynamically favorable—except for **108^Ts^**, which did not follow this trend. The results suggest that incorporating a strong electron-withdrawing group (CN) at the 6-position, alongside an electron-donating group (NMe_2_) at the 5′-position, enhances the strength of the intramolecular hydrogen bond. However, the replacement of the cyano group with NMe_2_ at the 5′-position lowers the PT barrier, further promoting ESIPT [187,188].

However, the authors do not specify any limitations in terms of bioimaging, such as photobleaching or chemical stability in the excited state, despite the absorption maxima of the developed biological fluorophores located in the blue region (up to 400 nm). Nonetheless, the possibility of introducing different groups into the 2-amino-pbt core allows for tuning the photophysical properties, making these compounds promising candidates for biological studies. It is possible that in the near future, 2-amino-pbt derivatives could become actively used bio-tools.

### 4.3. Applications in Catalysis

**Scheme 18:**
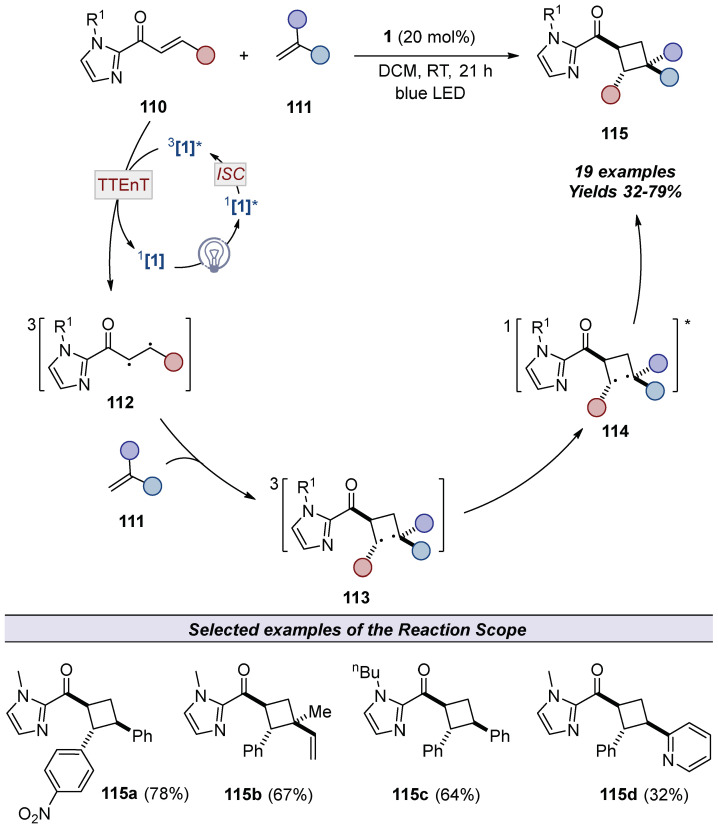
[2+2] photocycloaddition reaction using **1** as organic photocatalyst and proposed mechanism [90], [1]* is the photocatalyst in the excited state.

Among all catalysts reported in the literature, only several examples of Ir(III), Rh(III), and Pd(II) complexes with ***pbt*** derivatives have been described. These complexes have been used in cycloadditions [189,190], asymmetric Friedel–Crafts reactions, Michael additions, oxidation of alcohols [191], dehalogenation of aryl bromides [192], water reduction [43], and asymmetric transfer hydrogenation [193,194,195]. Until recently, **1** and its derivatives had not been used in catalysis. However, new visible-light organic photocatalysts based on the **1** framework have been developed for [2+2] photocycloadditions based on triplet-triplet energy-transfer (TTEnT) processes [90]. TTEnT covers a range of important chemical processes such as isomerization (*E/Z*, cyclopropanes, allenes, sulfoxides) [196], deracemization [197], photocycloaddition reactions ([2+2], [4+4]) [198,199,200,201] or homolytic σ-bond cleavage [202]. Particularly, the reaction of [2+2] photocycloaddition is an important tool in organic chemistry, which allows to synthesize products via the formation of two or several new bonds in one reaction step to obtain a variety of organic compounds with selective structure [203,204]. In a recent study [90], the catalytic properties of 2-RNH-pbt were explored in the reaction of cinnamyl imidazole **110** with a large excess of styrenes **111** (10 equiv.), giving **115** (Scheme 18). The proposed catalytic cycle for this [2+2] photocycloaddition reaction was suggested as follows (Scheme 18). Irradiation of the photocatalyst **1** at 450 nm would lead to the formation of the excited state ^1^[1]* of the photocatalyst, followed by intersystem crossing (ISC) to the triplet excited state ^3^[1]*. The latter would undergo triplet-triplet energy transfer with substrate **110,** providing an excited 1,4-biradical state **112**. Subsequent addition of the olefin **111** would afford **113,** and intersystem crossing would lead to the open-shell singlet **114**. Finally, radical recombination gives the cyclobutane product **115**. By varying the substituents on the amino-group in 2-RNH-pbt, it was demonstrated that the catalytic efficiency of ***pbt*** derivatives depends on the compounds’ light-absorbing properties and their tendency to undergo the ESIPT process. The triplet energy of 2-RNH-pbt compounds was calculated using TD-DFT, revealing that compounds have compatible triplet levels for the transfer of triplet energy to cinnamyl imidazole **110** under visible-light irradiation (450 nm). Photocatalysis was shown to proceed through the noncovalent interaction and triplet–triplet energy transfer between photocatalyst and substrate, as confirmed by NMR spectroscopy, time-resolved luminescence studies, and DFT calculations. A series of cyclobutane products with different functional groups were obtained in moderate to good yields, indicating that 2-NHR-pbt is a promising class of organic photosensitizers [90].

In summary, several potential applications have been discussed (i.e., as small molecule sensors, in APIs, in biosensing, and in catalysis). However, the practical implementation of these compounds is still in the early stages. We hope this review will encourage further interest in the active investigation of **1** and its derivatives, with practical applications remaining a goal for future research.

## 5. Conclusions

This review highlights the many synthetic possibilities to access 2-(2′-aminophenyl)benzothiazole **1** and its derivatives as well as their increasingly studied applications in coordination chemistry, in material sciences, for instance, in OLED technology, in the detection of cations, anions, and small molecules, in bioimaging, in the development of active pharmaceutical ingredients, and even in catalysis.

Compound **1** is a promising fluorescent core belonging to the NH-type of ESIPT systems. Although the intramolecular hydrogen bond in NH-type compounds is weaker than in other ESIPT systems, its main advantage is the ease of tuning the ESIPT rate. In this context, one can manipulate the relative probability of two processes (‘normal’ and ESIPT radiation decay) by external stimuli, thus tuning the emission color. Further studies of benzothiazoles may use the reviewed results to design molecular switches based on altered emission mechanisms.

The coordination chemistry of **1** and its derivatives has been actively studied since 2000 but is still limited to a small amount of different transition and f-elements. Based on SCXRD data, aminopbt derivatives typically coordinate in a chelate manner (i.e., N^3^ and N^2′^), however, other coordination modes are also envisageable. This makes coordinating chemistry of **1** and its derivatives an interesting area for further studies, including the use of other metal centers, the preparation of heterometallic compounds, and applications in catalysis. In terms of luminescent applications, incorporation of an ESIPT-active ***pbt***-based molecule in a coordination sphere of a metal ion seems promising since the resulting complex would feature one more radiation pathway, viz. phosphorescence while keeping the ESIPT site. The spectral profile of the luminescence, and consequently the emission color, can be varied by controlling the probability of three possible pathways: ‘normal’ fluorescence, ESIPT fluorescence, or phosphorescence. Compared to purely organic derivatives, the properties of such metal coordination compounds are much less explored, and thus more attention may be given to their design, including the study of the relationship between the emission processes.

## Figures and Tables

**Table 1:**
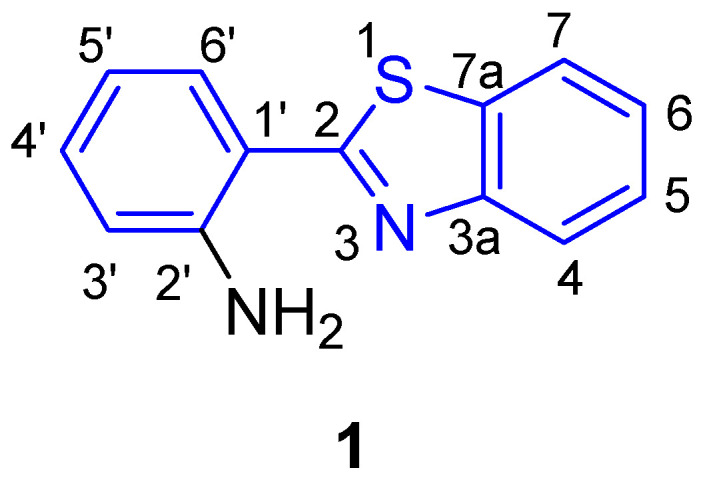
^1^H and ^13^C NMR data of **1** in different deuterated solvents [87].

^1^H NMR Chemical Shifts, ppm					^13^C NMR Chemical Shifts, ppm				
^1^H	Benzene	Chloroform	Acetone	DMSO	^13^C	Benzene	Chloroform	Acetone	DMSO
**3′**	6.32	6.78	6.92	6.90	**1′**	115.7	115.2	115.0	113.1
**4′**	7.00	7.22	7.22	7.22	**2′**	147.8	146.7	148.9	147.6
**5′**	6.55	6.75	6.68	6.65	**3′**	117.3	116.7	117.6	116.5
**6′**	7.70	7.71	7.69	7.63	**4′**	132.1	131.5	132.8	131.7
**4**	7.88	7.97	7.99	8.00	**5′**	117.1	116.8	116.9	115.6
**5**	7.12	7.45	7.50	7.50	**6′**	131.0	130.3	130.9	129.9
**6**	6.97	7.35	7.41	7.41	**2**	170.2	169.2	170.2	168.8
**7**	7.40	7.86	8.02	8.07	**3a**	154.7	154.7	154.8	153.2
**NH_2_**	6.15	6.40	7.11	7.34	**4**	123.0	122.4	123.1	122.0
					**5**	126.6	125.9	127.2	126.3
					**6**	125.3	124.8	125.4	125.0
					**7**	121.7	121.1	122.3	121.7
					**7a**	134.0	133.2	133.9	132.4

**Table 2:**
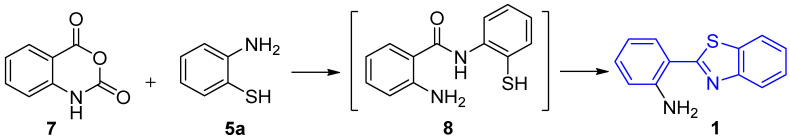
Synthesis of **1** using isatoic anhydride **7** as a starting material.

Entry	Reaction Conditions	t, °C	Time, h	Yield, %	Ref.
1	acidic kaolinitic clay (20% *w*/*w*), dried PhCl, argon atmosphere	120	20	55	[95]
2	CH_3_COONa, glacial acetic acid	120	3	80	[96]
3	ZnCl_2_ (30 mol%), PhCl	140	5	68	[97]

**Table 3:**
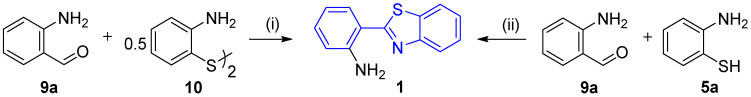
Synthesis of **1** using 2-aminobenzaldehyde **9** as a starting material.

Entry	Reaction Conditions	t, °C	Time, h	Yield, %	Ref.
(i)	modified silica dioxide catalyst (cat.), H_2_O, O_2_, I_2_	100	4	89	[102]
(ii)	CH_3_COOH, MW (490 W)	-	10 min	99	[103]

**Table 4:**
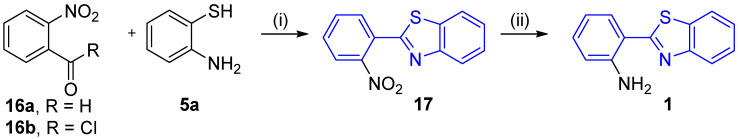
Two-step sequential synthesis of **1**, involving (i) formation and (ii) reduction of 2-(2′-nitrophenyl)benzothiazole **17**.

Entry	Starting Reagent	Step	Reaction Conditions	Time, h	Yield, %	Ref.
1	**16a**	(i)	K_3_[Fe(CN)_6_], toluene, reflux	10	72	[107]
		(ii)	NH_2_NH_2_·H_2_O, Pd-C, MeOH, reflux	8	85	
2	**16a**	(i)	640 W	10 min	78	[28]
		(ii)	SnCl_2_·2H_2_O, abs. EtOH, 70 °C	1	70	
3	**16a**	(i)	EtOH, 80 °C	3	71	[108]
		intermediate step	chloranil, CH_2_Cl_2_	overnight	86	
		(ii)	Fe, HCl, NH_4_Cl, THF/H_2_O, 60 °C	0.5	85	
4	**16b**	(i)	pyridine, r.t.	1	82	[106]
		(ii)	SnCl_2_·2H_2_O, EtOH, reflux	4	85	

**Table 5:**
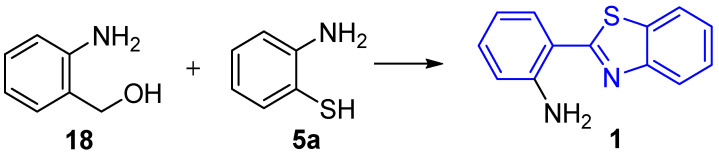
Synthesis of **1** using 2-aminobenzyl alcohol **18** as a starting material.

Entry	Reaction Conditions	t, °C	Time, h	Yield, %	Ref.
1	NaO^t^Bu (50 mol %), air, toluene	100	24	43	[109]
2	Pd(II) catalyst (1 mol%), m-xylene, KOH	120	12	71	[110]

**Table 6:**
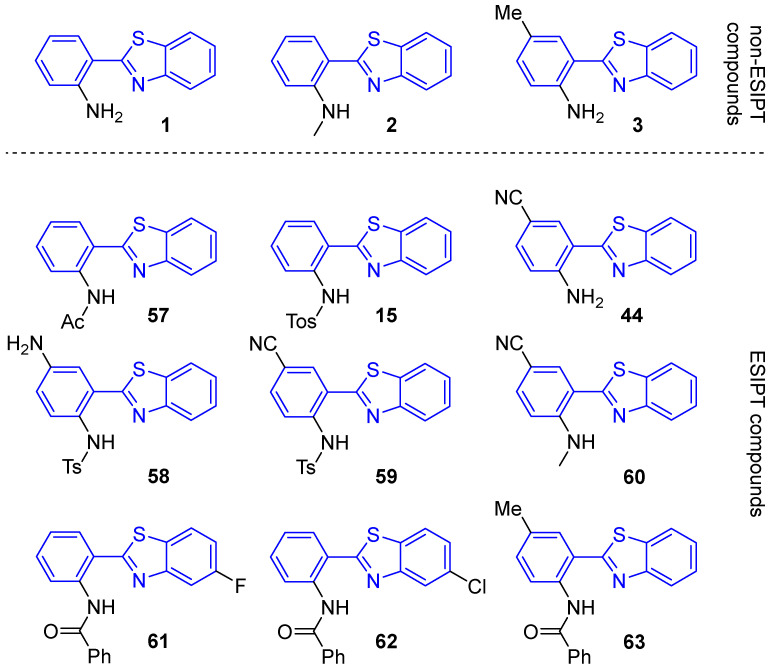
ESIPT and non-ESIPT compounds derived from **1** and their photophysical properties in solid state and/or in CH_2_Cl_2._

Compound	λ abs, nm	λ em, nm	T_S1_→T_S0_, nm	Ref.
**1**	360	455	-	[145]
**2** ^1^	392	458	-	[97]
**3**	392 *	469	-	[76]
**57** ^1^	335	395	565	[97]
**15** ^1^	330	-	555	[97]
**44** ^1^	363	419	588	[97]
**58** ^1^	362	-	649	[97]
**59** ^1^	336	-	540	[97]
**60** ^1^	393	436	623	[97]
**30e**	n.d.	420	550	[62]
**30f**	n.d.	435	550	[62]
**30g**	n.d.	440	565	[62]

^1^ data are presented for solution in CH_2_Cl_2_, *—compound absorbs in whole visible spectrum (400–800 nm).

**Table 7:**
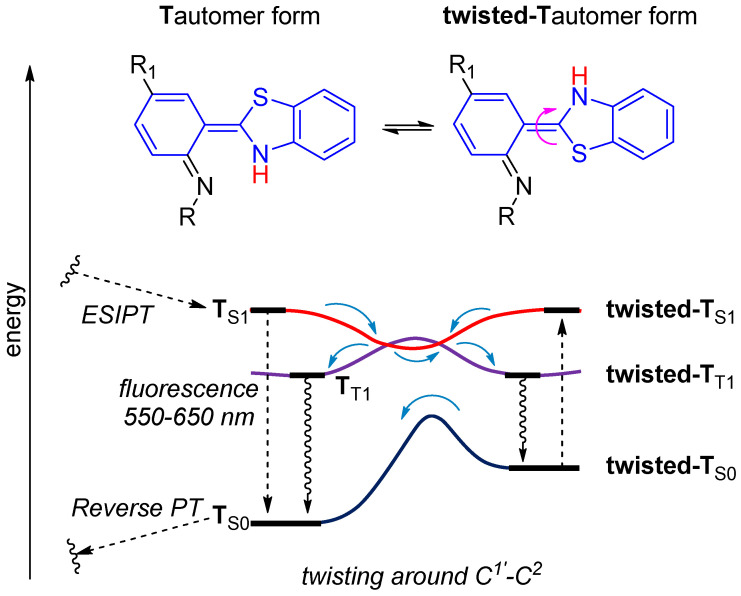
The calculated barriers for ESIPT (N_S1_→T_S1_) and twisting of tautomer form around C^1′^-C^2^ [148,149].

Compound	N_S1_→T_S1_, eV	Twisted-T_S0_→T_S0_, eV
**1**	0.38	not calculated
**2**	0.39	not calculated
**57**	0.30	0.33
**15**	0.12	0.27
**44**	0.34	0.34
**59**	0.12	0.83

**Table 8:**
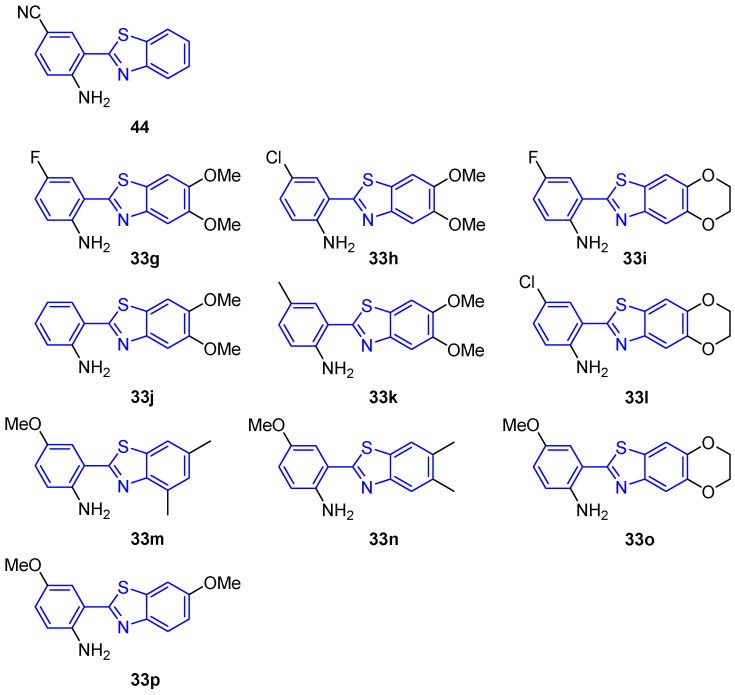
Luminescent properties of ***pbt*** derivatives with donor substituents in ***bt*** cycle in chloroform solution.

Compound	λ abs, nm	λ em, nm	Ref.
**44** ^1^	363	419, 588	[97]
**33g**	265, 378	440	[114]
**33h**	267, 379	440	[114]
**33i**	270, 306, 378	440	[114]
**33j**	265, 374	425	[114]
**33k**	265, 314, 377	440	[114]
**33l**	244, 270, 380	440	[114]
**33m**	303, 385	470	[114]
**33n**	300, 377	470	[114]
**33o**	245, 336, 390	470	[114]
**33p**	270, 322	465	[114]

^1^ data are presented for solution in CH_2_Cl_2._

**Table 9:**
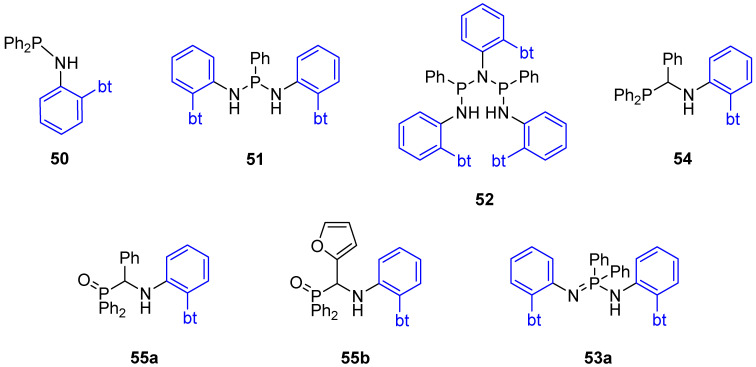
Photophysical properties in solid state of known organic phosphorus derivatives of **1**.

Compound	λ_abs_, nm	λ_em_, nm	τ, μs	Ref.
**50**	255, 263, 291, 330–374 (br)	455, 550	83	[82]
**51**	253, 262, 292, 307, 340–384 (br)	450, 575	212	[82]
**52**	254, 261, 284, 296, 316–379 (br)	455, 555	33, 700	[82]
**54**	260−320 (br.), 390	445, 600	-	[50]
**55a**	300, 380	450, 610	-	[83]
**55b**	300, 380	450, 610	-	[83]
**53a**	-	475, 634	-	[84]

**Table 10:**
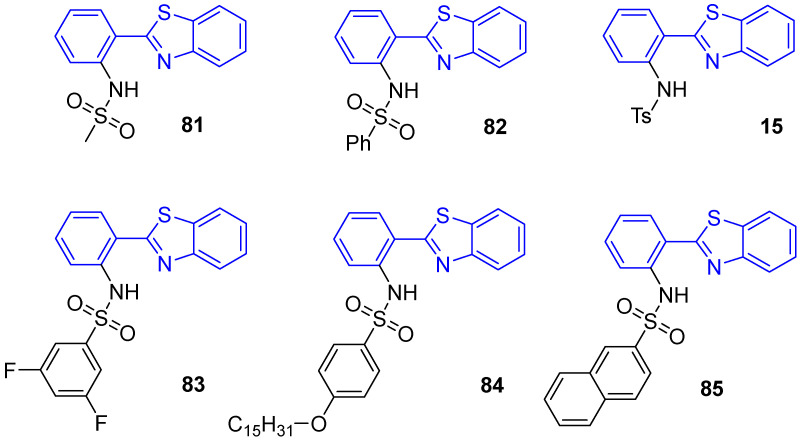
2-RNH-pbt proligands for Zn complexes with applications in OLED devices [164,165,166,167].

Compound	λ_abs_, nm	λ_em_, nm	Brightness *, cd·m^−2^ (at 8 V)
Zn(L**^81^**)_2_ (**86**)	385	463	100
Zn(L**^82^**)_2_ (**87**)	380	445	230
Zn(L**^15^**)_2_ (**88**)	390	440	140
Zn(L**^83^**)_2_ (**89**)	390	430	-
Zn(L**^84^**)_2_ (**90**)	380	445	270
Zn(L**^85^**)_2_ (**91**)	380	440	-

*—data are presented for devices based on corresponding complexes.

**Table 11:**
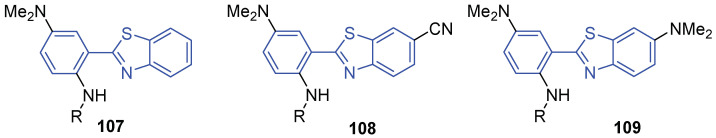
Calculated ESIPT barriers for 2-amino-pbt derivatives [188].

Compound	R = Ac, ESIPT Barrier, eV	R = Ts, ESIPT Barrier, eV
**107**	0.23	0.08
**108**	0.33	0.13
**109**	0.34	0.07

## Abbreviations

2-NH_2_-pbt	2-(2′-aminophenyl)benzothiazole
abs	Absorption
ACQ	Aggregation-caused quenching
AIE	Aggregation-Induced Emission
Ala^−^	Alaninate
Alk	Alkyl substituent
Ar	Aryl substituent
bt	2-benzothiazole
CBP	4,4′-bis(N-carbozolyl)-1,1′-biphenyl
CCDC	Cambridge Crystallographic Data Center
COD	Cycloocta-1,5-diene
Cp*	1,2,3,4,5-pentamethylcyclopentadienyl
DBU	1,8-Diazabicyclo(5.4.0)undec-7-ene
DCE	1,2-dichloroethane
DFT	Density-functional theory
DMI	Dimethyl-2-imidazolidinone
DMSO	Dimethylsulfoxide
DNA	Deoxyribonucleic acid
E	Element
em	Emission
equiv.	Equivalent
ESIPT	Excited State Intramolecular Proton Transfer
Fur	Furfuryl
Gly^−^	Glycinate
HEPES buffer	(4-(2-hydroxyethyl)-1-piperazineethanesulphonic acid)
het	Heterocyclic fragment
IR	Infrared
ISC	Intersystem crossing
IUPAC	International Union of Pure and Applied Chemistry
L	Ligand
LED	Light emitting diode
LLCT	Ligand-to-ligand charge transfer
LMCT	Ligand-to-metal-charge-transfer
Ln	Rare-earth metal
Mes	Mesityl
MLCT	Metal-to-ligand charge transfer
MW	Microwave irradiation
NIR	near-infrared region
NMR	Nuclear magnetic resonance
NPD	N,N’-di(1-naphthyl)-N,N’-diphenyl-(1,1′-biphenyl)-4,4′-diamine
NS0	Ground state of the normal form
NS1	Excited state of the normal form
OFED	Organic field-effect transistors
OLED	Organic light-emitting device
p-Tol	Para-tolyl group
pbt	2-phenylbenzothiazole
PET	Photoinduced electron transfer
PIC	Picrate ligand
PPA	Polyphosphoric acid
PPI	Pyrophosphate
PT	Proton transfer
PTA	Oligo(4,4′-(4″-methyl)triphenylamine)
RPT	Reverse proton transfer
S_1_	First singlet excited state
SCXRD	Single crystal X-ray diffraction
T_1_	First triplet excited state
TD-DFT	Time-dependent density-functional theory
TICT	Twisted intramolecular charge transfer
Tos	Tosyl
T_S0_	Ground state of the tautomer
T_S1_	Tautomer excited state
TTEnT	Triplet–triplet energy-transfer
